# Reach and engagement in the ECAIL trial targeting socially disadvantaged families across the COVID-19 pandemic and cost-of-living crisis: A mixed-methods implementation study

**DOI:** 10.1371/journal.pone.0350372

**Published:** 2026-10-08

**Authors:** Delphine Poquet, Camille Le Gal, Pascale Hincker, Laurent Béghin, Dominique Deplanque, Damien Subtil, Oriane Sion, Benjamin Cavalli, Lucie Vanhoutte, Victoria Jacobsen, Ketevan Marr, Ioannis Sakellaris, Blandine de Lauzon Guillain, Marie-Aline Charles, Delphine Ley, Priscille Sauvegrain, Sandrine Lioret

**Affiliations:** 1 Université Paris Cité and Université Sorbonne Paris Nord, Inserm, INRAE, Centre for Research in Epidemiology and Statistics, Paris, France; 2 Université Lille, Inserm, CHU Lille, Clinical Investigation Center, CIC 1403, Lille, France; 3 Université Lille, Inserm, CHU Lille, INFINITE - Institute for Translational Research in Inflammation, Lille, France; 4 Université Lille, Women and Newborn Mother Unit, CHU Lille, EA 2694, METRICS, Evaluation of Health Technologies and Medical Practices, Lille, France; 5 Research and Content Department, Association Programme Malin, Paris, France; 6 Division of Gastroenterology, Hepatology, and Nutrition, Department of Pediatrics, CHU Lille, Lille, France; 7 Universitary Department of Midwifery, Sorbonne Université, Paris, France; University of Huelva: Universidad de Huelva, SPAIN

## Abstract

**Background:**

The ECAIL trial, launched in 2017, was designed to evaluate a multicomponent intervention for childhood obesity prevention among hard-to-reach families. At a maternity hospital in Lille, France, healthcare providers screened pregnant women experiencing social vulnerability, and dietitians delivered a home-based intervention until age 2. The COVID-19 pandemic led to a six-month suspension in 2020. This study compared eligibility and participation before the pandemic and after resumption, and examined how implementation and reach evolved across the pandemic and subsequent cost-of-living crisis.

**Methods:**

Using a convergent mixed-methods design, we analyzed 5,744 eligibility questionnaires distributed at the maternity ward and conducted a focus group with six dieticians delivering the intervention to triangulate quantitative implementation indicators with qualitative implementation data. Inclusion criteria included ≥1 indicator of social vulnerability (e.g., socioeconomic disadvantage, precarious housing, or social isolation). The focus group was recorded, transcribed, and analyzed thematically focusing on reach, acceptability, and adaptation.

**Results:**

Eligibility increased from 29.7% (n = 955) pre-pandemic to 33.6% (n = 849) after resumption, while the distribution of vulnerability criteria remained similar across periods: 78.3% received social/medical benefits; employment was not the main source of household income for 58.7%; 24.4% experienced financial hardship; 14.7% reported social isolation; 6.0% lived in precarious housing; and 19.0% had three or more vulnerabilities. Participation among eligible women remained stable (24.6%; n = 443). Qualitative findings indicated dietitians’ satisfaction and participants’ enthusiasm for the resumption of home visits, particularly in addressing social isolation. After resumption, the introduction of a pre-visit COVID-19 questionnaire was associated with fewer failed home visits. Integration of quantitative and qualitative findings indicated sustained, and in some cases strengthened, provider engagement despite pandemic-related strain on hospital services.

**Conclusions:**

This study suggests that a complex intervention can maintain reach and acceptability through adaptive implementation under major contextual disruptions. The rapid resumption of home-based services emerged as a promising implementation strategy for engaging and retaining socially disadvantaged families, highlighting the importance of flexible, context-sensitive approaches during social and economic crises.

**Trial registration:**

Clinicaltrials.gov NCT03003117; registration date: 21/12/2016; https://clinicaltrials.gov/study/NCT03003117.

## Introduction

In 2020, the COVID-19 pandemic triggered a massive health crisis worldwide, accompanied by prolonged economic and social repercussions. Following the initial lifting of pandemic-related health restrictions, subsequent geopolitical shocks, including the invasion of Ukraine, fueled inflation and rising living costs across Europe [1]. Although governments implemented exceptional support measures, low-income households experienced a disproportionate loss of purchasing power, particularly for food, transportation, and housing, further exacerbating pre-existing social, economic, and health inequalities [2–10]. These successive health and cost-of-living crises have therefore disproportionately affected populations already experiencing social disadvantage, illustrating a cumulative disadvantage often described as the “poverty penalty” [6].

Numerous studies have underscored the detrimental impact of the COVID-19 pandemic on health-related behaviors (such as physical activity, sedentary behaviors, and dietary intake), as well as on obesity and mental health in both adults and children [11–17]. Far less attention, however, has been given to how such large-scale disruptions have affected the implementation of ongoing public health interventions, particularly those targeting socially disadvantaged populations. From an implementation science perspective, understanding how implementation outcomes such as reach, engagement, and fidelity are sustained, adapted, or disrupted under crisis conditions remains a critical gap in the literature [18]. Frameworks such as RE-AIM highlight the importance of examining reach, adoption, and implementation processes alongside effectiveness, particularly for interventions targeting socially disadvantaged groups [19,20] that are considered “hard-to-survey”, that is, populations who are not only difficult to sample, but also challenging to identify, reach, recruit, engage, and retain over time [21,22].

We seek to address this gap by analyzing multiple sources of evidence generated within the prEgnanCy and eArly Childhood nutrItion triaL (ECAIL), launched in 2017 in northern France [23], a region characterized by high levels of socioeconomic disadvantage [24]. The overarching aim of ECAIL is to assess the effectiveness of a multilevel and multibehavioral intervention initiated during pregnancy that combines nutritional counseling with facilitated access to healthy foods at a reduced price, with the goal of promoting healthy lifestyle behaviors and preventing obesity in young children from underserved families. Building on an interdisciplinary implementation science approach, this study adopts a convergent mixed-methods design to triangulate quantitative and qualitative findings and provide complementary insights into trial implementation [25]. Consistent with the RE-AIM framework, the present implementation study focuses specifically on the Reach, Adoption, and Implementation dimensions, whereas Effectiveness and Maintenance dimensions are beyond its scope and will be reported separately. Specifically, we aimed to (i) describe and compare eligibility and participation rates before the pandemic and after the trial resumed six months later, with particular attention to reach and its social patterning, and (ii) explore implementation processes, including delivery adaptations and stakeholder engagement, across the pandemic and subsequent cost-of-living crisis.

## Methods

### Trial design and participants

ECAIL is a single-blind randomized controlled trial launched by the French National Institute of Health and Medical Research (Inserm) in 2017 in northern France to evaluate the effectiveness of an existing program, called *Malin*, delivered by trained dietitians [23]. Beyond its evaluative design, ECAIL is also a participatory action research project [26], co-created with members of the *Malin* nonprofit association and its historical stakeholders, namely the French Red Cross, the French Society of Pediatrics, and the French Association for Ambulatory Pediatrics, in close collaboration with investigators from the Lille University Hospital and the Valenciennes Hospital. This co-creation process aimed to enhance the intervention’s contextual relevance, feasibility, and acceptability for socially disadvantaged families, as well as its integration into existing care pathways. More details about the ECAIL trial design, intervention components, and implementation procedures are provided in the published protocol [23].

Pregnant women were screened for eligibility during routine prenatal care at the maternity wards of Lille University Hospital (from March 2017 to April 2023; Fig 1), where 443 women were enrolled, and at Valenciennes Hospital. This analysis focuses exclusively on the Lille site, as the Valenciennes center opened in June 2021, more than one year after the onset of COVID-19.

**Fig 1:**
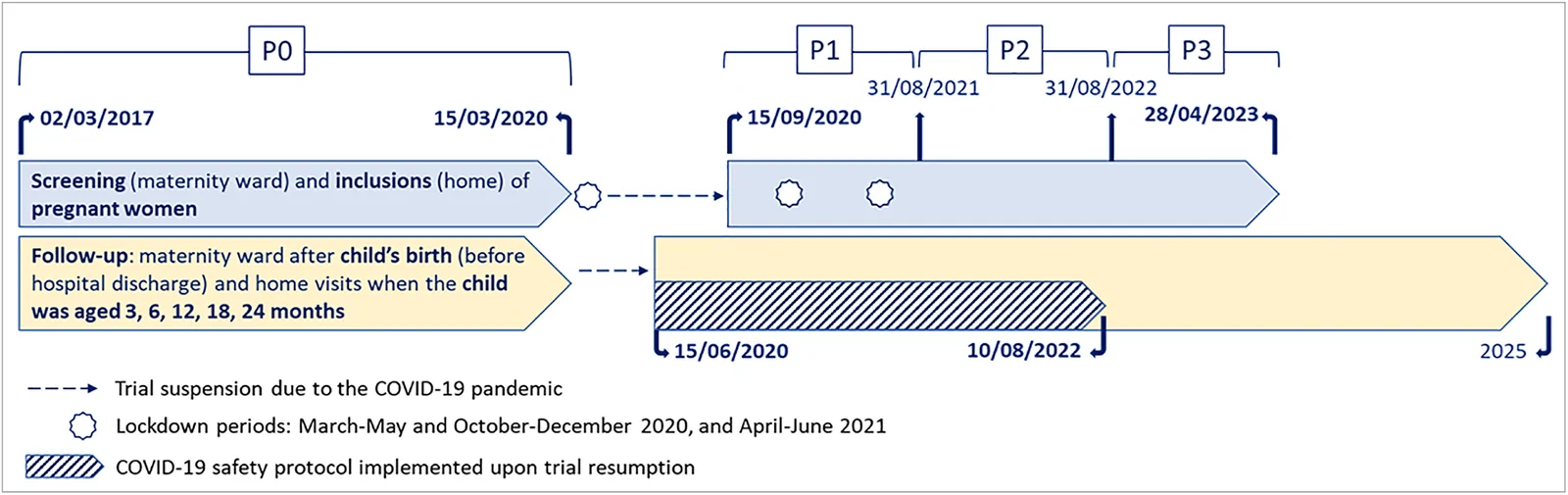
Timeline of ECAIL trial implementation in the Lille study area. The ECAIL trial.

While waiting for their prenatal appointment, pregnant women were given an eligibility questionnaire (S1 File) by reception staff, covering all inclusion and exclusion criteria (Table 1). Some forms of precarious housing, such as substandard living conditions, were considered as inclusion criteria while more unstable forms of housing precarity such as homelessness or multiple moves in the last 12 months were considered as exclusion criteria, in order to maximize the likelihood of longitudinal follow-up.

**Table 1:** Inclusion and exclusion criteria of the ECAIL study, which the eligibility questionnaire screened for. The ECAIL trial.

Inclusion criteria	Exclusion criteria
• Pregnant woman at 3^rd^ trimester of pregnancy, with health insurance coverage (national health insurance fund or state medical assistance); • Age ≥ 18 years; • Lives in Lille, Valenciennes, or the surrounding areas; • Willing and able to sign informed consent; • Who, in the Investigator's judgment, is able to comply with the protocol and study procedures (including speaking French); • Who currently faces social disadvantages, defined by the presence of ≥1 of the following social vulnerabilities: ◦ receives social or medical benefits; ◦ the household's main source of income is not employment (but rather social benefits, friends/family, or none); ◦ perceived financial hardship, i.e., experiences financial hardship in meeting her needs, such as food, medication, rent, and bills; ◦ precarious housing, i.e., sheltered in a long-term rehabilitation center or in a social hotel; or lack of any of the following facilities: shower or bath, toilet, running water or heating system; ◦ social isolation, i.e., planning to look after the baby on her own after birth, without a partner or another adult to help.	• Pregnant woman under legal guardianship; • Participation in any other interventional clinical trial that is not compatible with the participation into the ECAIL study; • No permanent mailing addresses; housed in emergency accommodation centers, hotels, portable dwellings or homeless; • Unstable housing (has moved several times in the past 12 months or planning to move outside the study area before the child's first birthday); • Serious illness during pregnancy that could interfere with optimal participation in the ECAIL study; • Illegal substance drug use (excluding cannabis); • Multiple pregnancy (≥3 fetuses).

A prescreening phase was implemented by the ECAIL dietitians to avoid the distribution of the eligibility questionnaire to pregnant women living outside the study area; aged <18 years; before 4 and after 7 months of gestation; or enrolled in another trial not compatible with participation into the ECAIL study.

During the prenatal appointment, healthcare providers (HCPs; e.g., midwives, nurses, or obstetricians) used this questionnaire to determine eligibility for the ECAIL trial. When a woman was deemed eligible, the HCP was expected, as part of the screening protocol, to contact a dietitian from the ECAIL implementation team by telephone. The dietitian then provided the woman and her partner (when present) with information about the study in person after the HCP appointment. Importantly, the provision of study information face to face by dietitians, rather than exclusively by telephone, served as an indirect indicator of HCP engagement with the screening procedure. Women were subsequently given time to consider participation. Within the following week, the same dietitian contacted the eligible woman by telephone to assess her willingness to participate. If she agreed, an appointment was scheduled at her home (or another location of her choice) for the baseline visit during the third trimester of pregnancy. If the eligible woman declined participation, the dietitian recorded her reasons on the eligibility questionnaire.

When the HCP failed to call an ECAIL dietitian, they transmitted the questionnaire at a later time, and a dietitian then contacted the woman by telephone to provide study information. Women were then offered a one-week reflection period, followed by an additional call to discuss participation. Moreover, as part of the co-creation process, the eligibility questionnaire was co-designed, pilot-tested, and iteratively refined with HCPs, and monthly coordination meetings were used to identify barriers and collectively develop solutions to support implementation.

During the baseline home visit, the mother-to-be provided written informed consent for the participation of the mother-child dyad, randomization was performed, and the inclusion process was formalized. Subsequently, participating families were followed up by dietitians, first at the maternity ward shortly after delivery, and then through five additional home visits until the child's second birthday, that is, at 3, 6, 12, 18, and 24 months.

Due to the COVID-19 pandemic, screening, inclusions, and home visits were suspended during the first national lockdown, which started on March 15, 2020 (Fig 1), when 140 families were actively under follow-up in the study. To enable the safe continuation of the trial, implementation adaptations were introduced upon resumption. Home visits (June 2020) and screening/inclusions (September 2020) resumed under an adapted delivery protocol. Dietitians contacted participants by telephone before each home visit to administer a questionnaire assessing COVID-19 symptoms within the previous three weeks and recent contact with a confirmed case. Dietitians had the option to consult a physician to decide whether to proceed with the visit or postpone it for two weeks. Before the pandemic, dietitians typically sent a text message prior to visits without requiring participant confirmation; this pre-visit telephone contact was introduced upon trial resumption. During home visits, dietitians were required to comply with protective measures, including wearing masks, practicing hand hygiene, and maintaining social distancing, and participants were requested to do the same. These adaptations allowed the trial implementation to continue while maintaining feasibility and in-person contact with families.

### Overall mixed-method design

This study adopted a convergent mixed-methods design [25]. Quantitative and qualitative data were collected and analyzed independently to examine complementary dimensions of implementation. Integration occurred during interpretation through triangulation of findings, with the aim of assessing the convergence, complementarity, or divergence between implementation indicators derived from routine trial data and dietitians’ experiences of intervention delivery.

### Data collection and measurements

#### Time periods.

We defined two periods, namely pre-pandemic (March 2017 to March 2020; P0) and post-study resumption (September 2020 through April 2023). As shown in Fig 1, the post-resumption period encompassed successive phases of the COVID-19 pandemic and the progressive relaxation of public health measures. These phases corresponded to successive changes in national public health restrictions and hospital infection-control procedures that characterized the implementation context of the ECAIL trial, including mandatory masking and the study-specific COVID-19 safety protocol introduced upon trial resumption. To capture temporal variation in implementation conditions, the post-resumption period was segmented into three approximately one-year periods (September-August). This segmentation also ensured comparable seasonal coverage across periods. The final period was shorter due to the end of screening in April 2023 (Fig 1; P1, P2, and P3).

#### RE-AIM indicators and their measurement.

Drawing on the RE-AIM framework, the following indicators were selected: eligibility, participation, and the social vulnerability profiles of eligible, ineligible, participating, and nonparticipating women as indicators of *Reach*; delivery modalities and follow-up completion and adaptations as indicators of *Implementation* [19,20]. The frequency of face-to-face delivery of study information was also interpreted as an indirect indicator of HCP engagement and, therefore, *Adoption*, because it required HCPs to actively contact an ECAIL dietitian during the participant screening procedure [19,20].

*Eligibility.* The eligibility rate was calculated as the number of eligible pregnant women (according to the responses collected from the eligibility questionnaires) divided by the number of eligibility questionnaires completed by pregnant women and returned to the ECAIL dietitians. As a proxy for implementation processes and provider engagement, the mode of study information delivery by dietitians to eligible women was categorized as either in person or by telephone.

*Participation*. The participation rate was calculated as the number of actual inclusions in the ECAIL trial divided by the number of eligible pregnant women. Non-participation was classified into four categories: women who were unreachable; screening failures (i.e., cases in which inclusion could not be completed, regardless of the women's decision, due to timing constraints, pregnancy outcomes, or unforeseen exclusion criteria); refusals without stated reasons; and refusals with one or more reasons, recorded verbatim by ECAIL dietitians.

*Follow-up.* Based on the theoretical number of follow-up visits expected to take place from trial commencement to the end of the screening in April 2023, we calculated the rate of skipped visits. We also calculated the rate of failed visits, defined as cases in which the dietitian found nobody at the participant’s home, expressed as the number of failed visits divided by the number of trips made by dietitians.

#### Indicators of social vulnerability.

Six binary variables (yes/no) were defined using information available in the eligibility questionnaire: receipt of social or medical benefits; household main income not derived from employment; perceived financial hardship; social isolation; precarious housing; and experiencing ≥3 of these social vulnerabilities. The latter indicator was defined as the sum of the first five social vulnerability variables, with “yes” coded as 1 and “no” as 0.

#### Focus group.

A researcher in psychosocial sciences (DP) conducted an in-person focus group in May 2023 at the Lille University Hospital with the complete ECAIL implementation team, comprising six dietitians (including the coordinator), to explore the impact of the COVID-19 pandemic on trial implementation. Written informed consent was obtained from each dietitian.

Several considerations guided the use of a focus group [27,28]. In line with the convergent mixed-methods design [25], the qualitative component was intended to complement and contextualize the quantitative implementation indicators through triangulation, rather than explain them sequentially. First, the COVID-19 pandemic substantially disrupted the usual implementation conditions, warranting an in-depth qualitative assessment alongside quantitative indicators. Second, dietitians were identified *a priori* as the most relevant stakeholders for this qualitative component, given their central role in trial implementation and their sustained engagement with participants throughout the study. Third, as the dietitians shared a common implementation context, a focus group facilitated the collection of shared perspectives, experiences, and adaptations. This approach was particularly suited to capturing local contextual influences on intervention delivery.

The ECAIL RCT has approval from the committee for protection of persons engaged in research (*Comité de Protection des Personnes Nord-Ouest 1,* CPP, 24/07/2014), the national advisory committee on information processing in health research (*Comité consultatif sur le traitement de l'information en matière de recherche,* CCTIRS, 04/09/2014), the national data protection authority (*Commission nationale de l'informatique et des libertés,* CNIL, 15/06/2016). The protocol for the qualitative component was approved by the CPP on 28/06/2021. Written informed consent was obtained from all participants.

### Analysis

#### Quantitative analysis.

The quantitative analysis primarily consisted of describing implementation-related indicators across study periods, in line with the RE-AIM framework. Where appropriate, formal statistical comparisons were performed to assess differences either between the two main study periods (pre-pandemic vs post-study resumption) or across the four study periods (P0–P3), depending on the analysis presented. In addition to the eligibility rate, we compared the following implementation-related indicators between the pre-pandemic and post-resumption periods:

- among eligible women: participation rate, distribution of social vulnerability indicators, and mode of delivery of study information (in person or by telephone);
- among nonparticipating women: distribution of the main reasons for not participating;
- among participating women: rates of both skipped and failed follow-up visits;
- among ineligible women: proportion presenting at least one indicator of social vulnerability, along with the distribution of their social vulnerabilities and exclusion criteria.

Two-sided χ2 tests were used for categorical variables.

All analyses were conducted using RStudio (2022) (RStudio: Integrated Development Environment for R. RStudio, PBC, Boston, MA).

#### Qualitative analysis.

DP manually transcribed the focus group verbatim and imported it into NVivo software. A thematic content analysis [29] was then conducted. The transcript was coded by DP, and codes were iteratively grouped into broader themes reflecting implementation context, disruptions, adaptations, and perceived mechanisms influencing reach and engagement. The coding and resulting themes were independently reviewed by PS, and any discrepancies were discussed until consensus was reached. Illustrative quotations were then selected to exemplify the identified themes and subthemes and reflect dietitians’ shared experiences of trial implementation during the pandemic.

## Results

Results are presented across three RE-AIM–relevant dimensions in disrupted contextual conditions, namely *Reach*, *Adoption*, and *Implementation*.

### Descriptive findings from the quantitative component

#### Eligibility for the ECAIL trial.

In all, 5,744 pregnant women completed the eligibility questionnaire between March 2017 and April 2023 at the Lille University Hospital maternity ward; 1,804 were deemed eligible for the ECAIL trial. Among the women screened, 29.7% (n = 955) were eligible in the pre-pandemic period and 33.6% (n = 849) post-resumption (*P* = 0.002, comparison between the two main study periods). The eligibility rate remained consistently higher across each of the three post-resumption subperiods (P1–P3), as illustrated in Fig 2.

**Fig 2:**
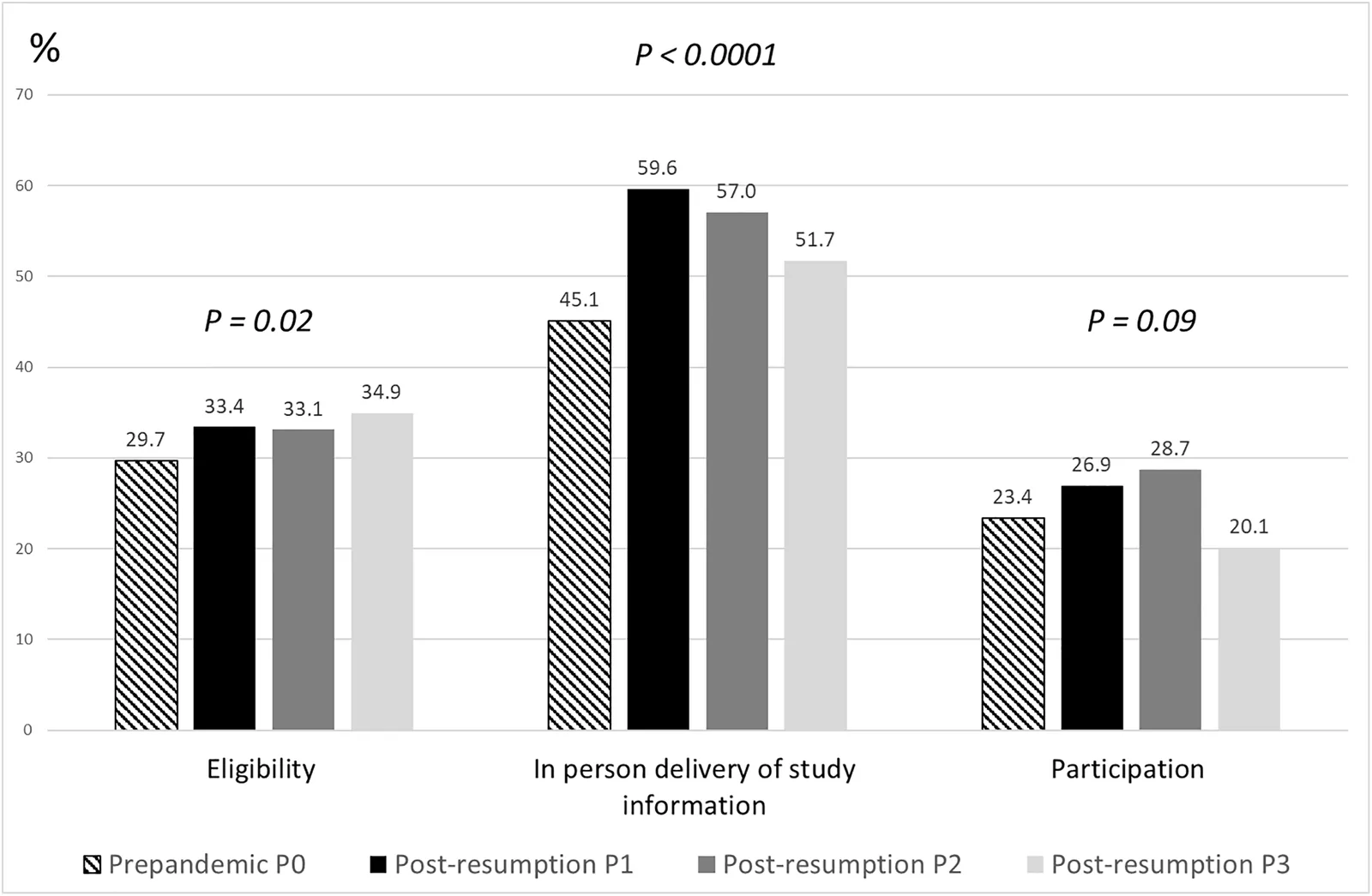
Rates of eligibility for the ECAIL trial (n = 1,804), and rates of in-person delivery of study information (n = 915) and participation (n = 443) among eligible women according to the four study periods; pre-pandemic, P0: March 2017 to March 2020; post-resumption, P1: September 2020 to August 2021; P2: September 2021 to August 2022; P3: September 2022 to April 2023. *p* value from χ² test comparing P0, P1, P2 and P3. The ECAIL trial, Lille study area.

Overall, no significant differences were observed in the distribution of social vulnerability indicators among eligible women between the pre-pandemic and post-resumption periods (Table 2): 78.3% received social or medical benefits; employment was not the main source of household income for 58.7%; 24.4% experienced financial hardship; 14.7% reported social isolation; 6.0% lived in precarious housing; and 19.0% presented three or more of these social vulnerabilities. However, when examined across post-resumption subperiods, Fig 3 shows that the highest observed proportion of eligible women for whom the main source of income in the household did not derive from employment occurred during P1 (64.8%), as did the receipt of social or medical benefits (82.8%). The highest observed proportion of women reporting financial hardship occurred during P3 (29.3%). Only 50.7% of eligible women received information on the study in person from the ECAIL dietitians, rather than by telephone, but this rate was higher post-resumption (57.0% compared with 45.1% before the pandemic, Table 2). Fig 2 shows that the highest observed rate occurred during P1 (59.6%) before decreasing to 51.7% in P3.

**Table 2:** Distribution of social vulnerabilities and study implementation indicators among eligible women by study period. The ECAIL trial, Lille study area.

	March 2017 to Apr 2023 % (n = 1,804)	March 2017 to March 2020 % (n = 955)	Sept 2020 to Apr 2023 % (n = 849)	*p*-value
**Social vulnerabilities**				
Receives social or medical benefits	78.3	77.2	79.5	0.23
Household's main source of income is not employment	58.7	56.8	60.9	0.07
Perceived financial hardship	24.4	25.8	23.0	0.17
Social isolation	14.7	14.3	15.1	0.66
Precarious housing	6.0	5.5	6.6	0.35
≥ 3 social vulnerabilities	19.0	18.8	19.2	0.81
**Study implementation indicators**				
*Screening*				
In-person delivery of study information	50.7	45.1	57.0	<0.0001
*Follow-up* ^*a*^				
Skipped visits^b^	2.0	1.7	2.1	0.49
Failed visits^c^	3.0	4.9	1.7	<0.001

*p*-values compare the pre-pandemic (March 2017–March 2020) and post-resumption (September 2020–April 2023) periods using χ² tests. ^a^ Follow-up among 443 participants, corresponding to 919 theoretical visits before the pandemic and 1,397 after study resumption. ^b^ The numbers of skipped visits were 16 before the pandemic and 30 after study resumption. ^c^ The numbers of failed visits (and corresponding trips made) were 47 (959) before the pandemic and 23 (1,390) after study resumption.

**Fig 3:**
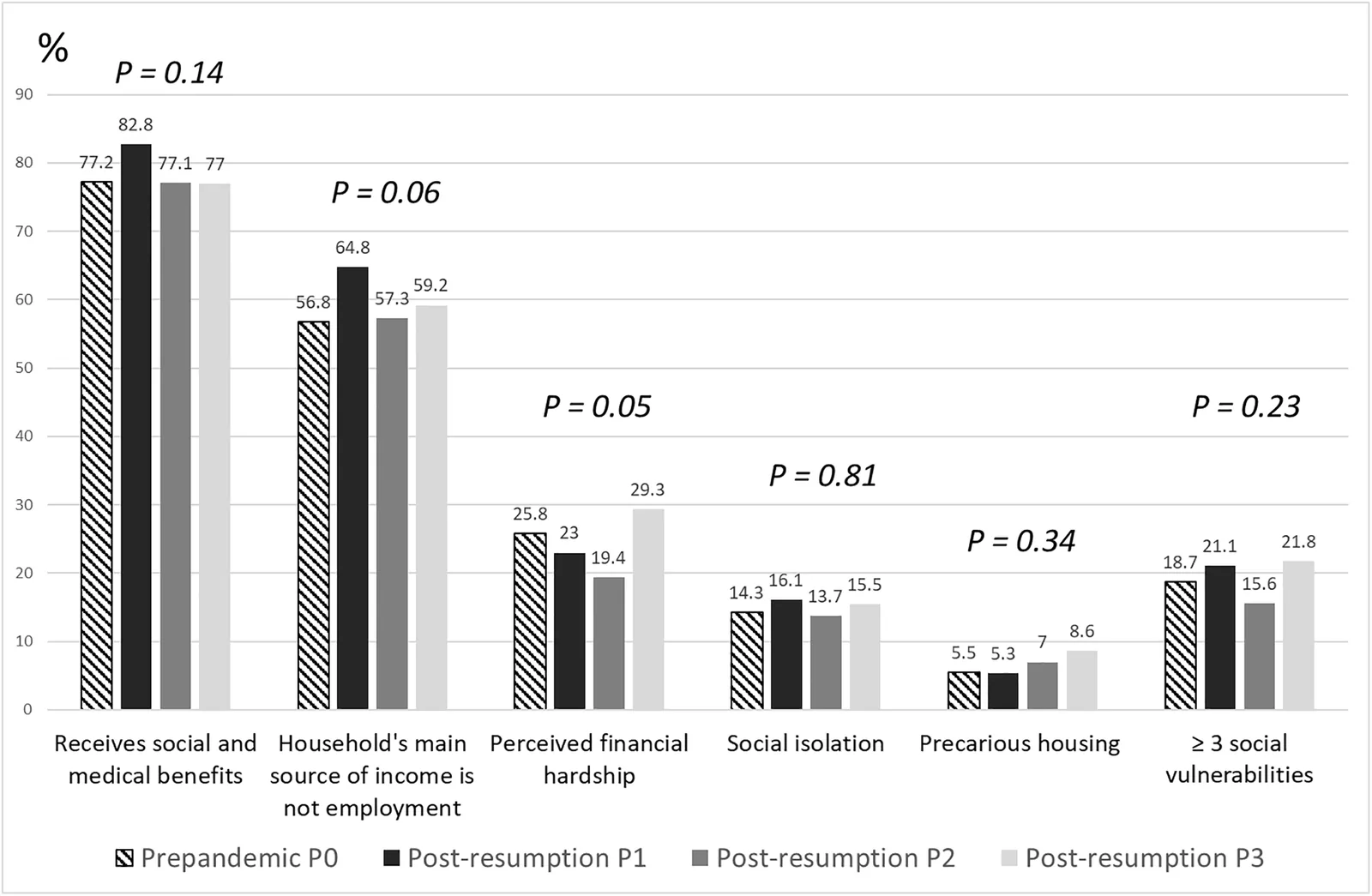
Distribution of social vulnerabilities among eligible women (n = 1,804) according to the four study periods; pre-pandemic, P0: March 2017 to March 2020; post-resumption, P1: September 2020 to August 2021; P2: September 2021 to August 2022; P3: September 2022 to April 2023. *p* value from χ² test comparing P0, P1, P2 and P3. The ECAIL trial, Lille study area.

#### Participation in the ECAIL trial.

Overall, 24.6% of eligible women consented to participate and were included in the trial, with no difference in participation rates between pre-pandemic and post-resumption periods. Fig 2 shows that participation was highest during P1 (26.9%) and P2 (28.7%) and lower during P3 (20.1%), falling below the pre-pandemic level observed in P0 (23.4%). The distribution of social vulnerability and implementation-related indicators differed between participating and nonparticipating women (Table 3). Across both periods, women who agreed to participate more frequently reported perceived financial hardship and were more often informed about the study in person compared with those who declined. One exception concerned precarious housing, for which the direction of the association with participation differed between the pre-pandemic and post-resumption periods.

**Table 3:** Characteristics of eligible women according to participation status and study period. The ECAIL trial, Lille study area.

	March 2017 – March 2020 % (n = 955)			Sept 2020 – Apr 2023 % (n = 849)		
	Participants	Non-participants	*p*-value	Participants	Non-participants	*p*-value
**Social vulnerabilities**						
Receives social or medical benefits	74.9	77.9	0.35	77.5	80.2	0.38
Household's main source of income is not employment	57.8	56.4	0.71	57.7	62.0	0.25
Perceived financial hardship	37.2	22.3	<0.0001	29.7	20.6	0.005
Social isolation	15.7	13.9	0.51	14.4	15.3	0.75
Precarious housing	7.2	5.1	0.23	3.2	7.8	0.02
≥ 3 social vulnerabilities	24.7	16.9	0.01	17.1	19.9	0.36
**Study implementation**						
In-person delivery of study information	54.3	42.3	0.002	64.9	54.2	0.006

Within each study period, *p*-values compare participants and non-participants using χ² tests.

#### Nonparticipation in the ECAIL trial, despite eligibility.

There was no major difference in the overall pattern of nonparticipation between the pre-pandemic and post-resumption periods: a majority of eligible women either remained unreachable or declined participation without providing a reason, while 29% gave at least one reason for nonparticipation (Fig 4). Screening failures primarily occurred when pregnancies were too advanced or births took place before the scheduled baseline visit, with the latter more frequent post-resumption, reflecting the suspension of the inclusion process during the first lockdown (Fig 5). Among women who provided reasons for nonparticipation, time and availability constraints were reported more frequently post-resumption, whereas partner or relative refusal and having older children were less common (Fig 6).

**Fig 4:**
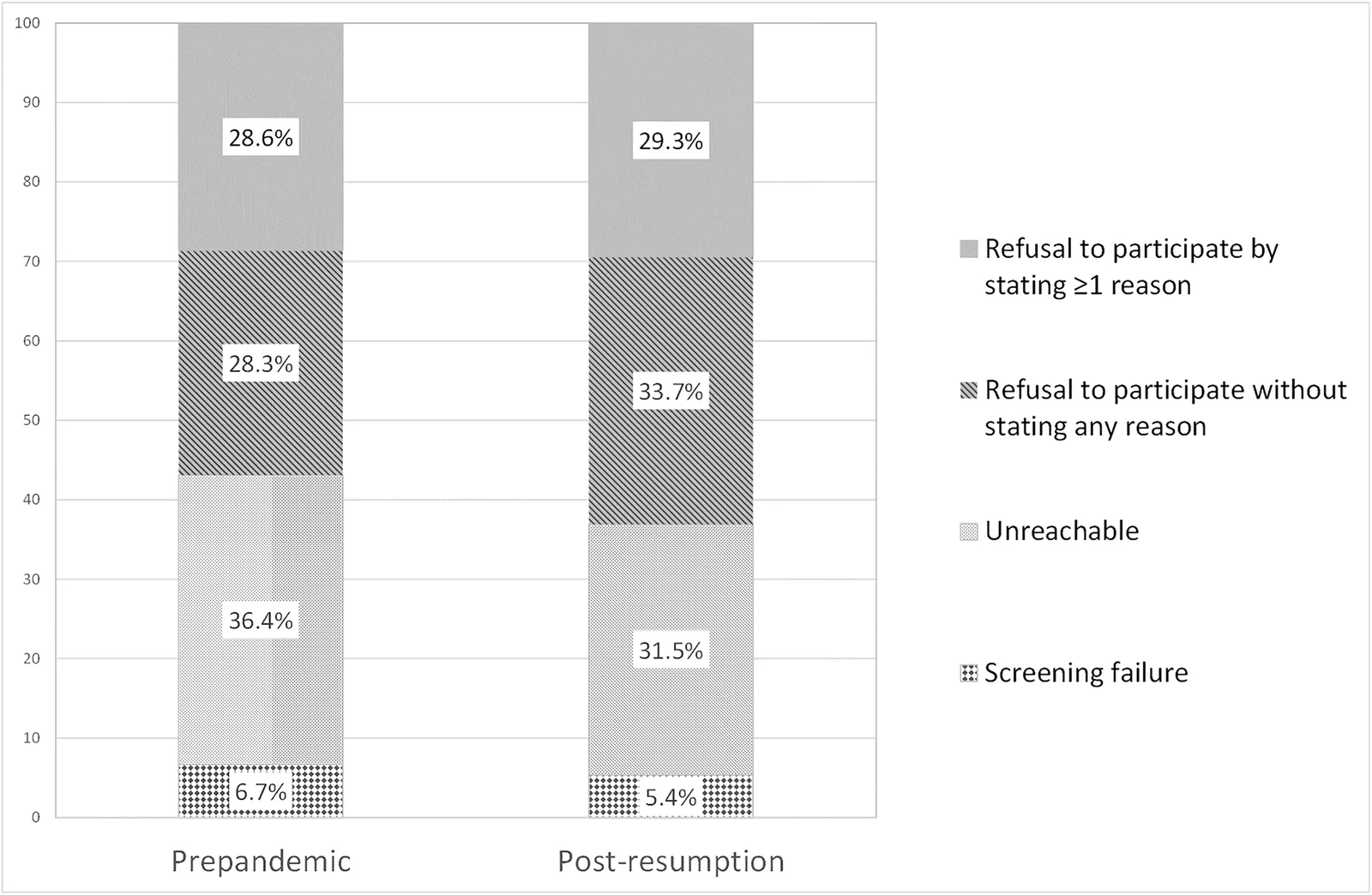
Distribution of the four main reasons for nonparticipation in the study among eligible nonparticipating women (n = 1,361) by period (pre-pandemic and post-resumption). The ECAIL trial, Lille study area.

**Fig 5:**
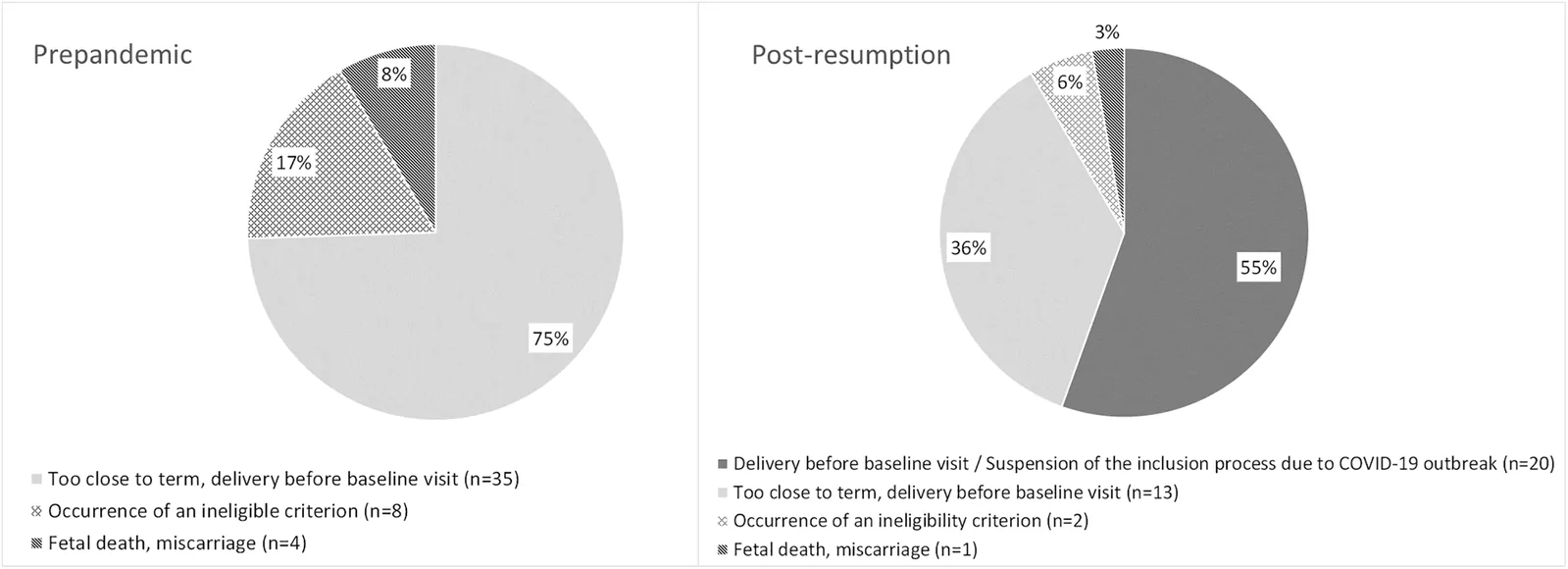
Characterization of screening failures by study period (pre-pandemic and post-resumption). The ECAIL trial, Lille study area.

**Fig 6:**
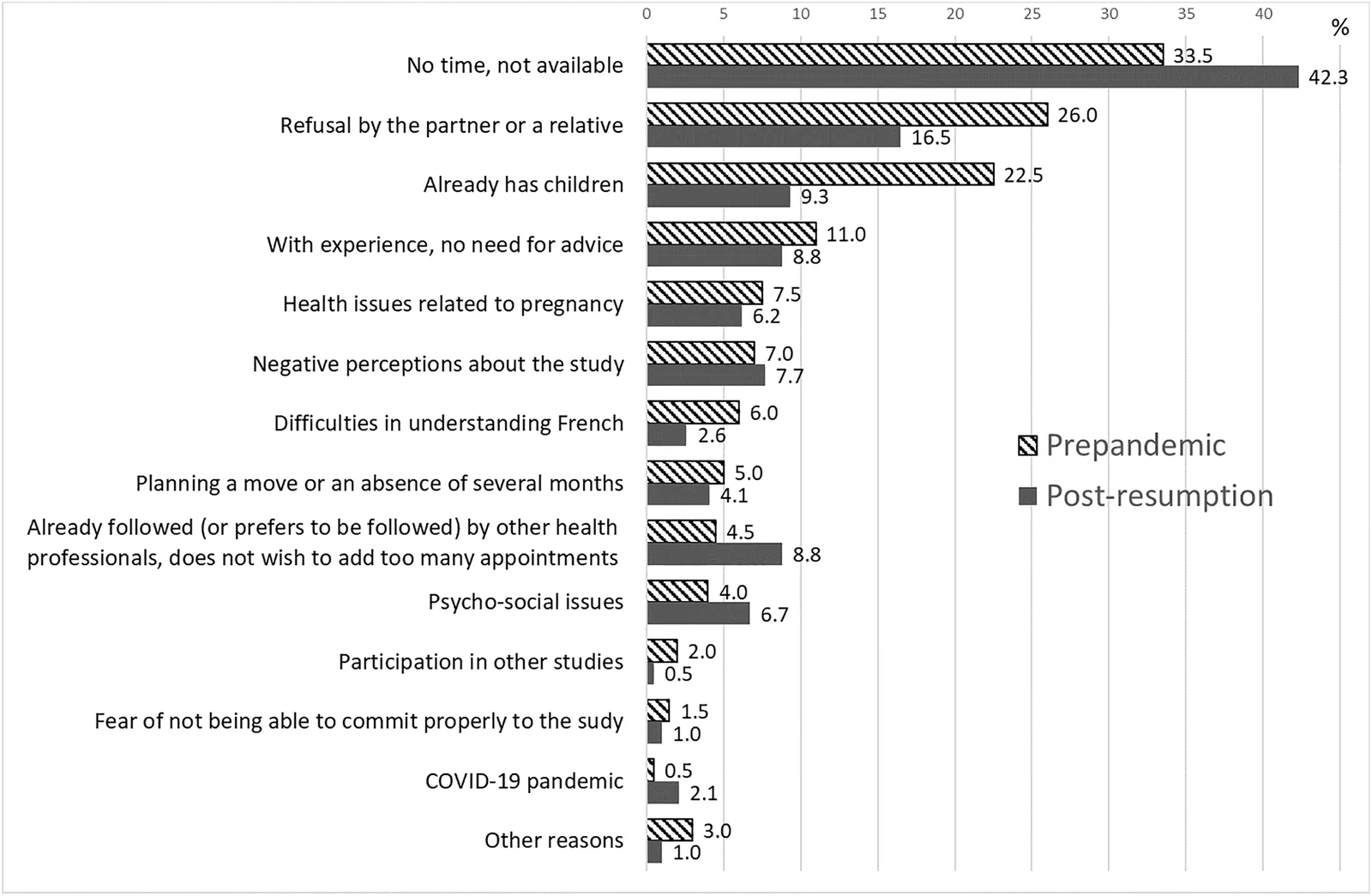
Distribution of the reasons for nonparticipation stated by 396 women by study period (pre-pandemic and post-resumption). **The ECAIL trial, Lille study area.** *Negative perceptions about the study* include “Too long”, “Too constraining”, “Not useful”; *Psycho-social issues* include “Personal reasons”, “Overwhelmed”, “Too many problems to deal with”, “Health issues with a child or another family member”, “Too instable”, “Inability to plan ahead”; *Participation in other studies* includes in the past or currently.

#### Ineligibility for the ECAIL trial despite social disadvantage.

Among ineligible women, 18.9% (n = 746) were identified as experiencing at least one social vulnerability criterion (according to the eligibility questionnaire) but also met at least one exclusion criterion. Their social vulnerability profile was overall more disadvantaged than that of eligible women, particularly with respect to unemployment, perceived financial hardship, precarious housing, and the accumulation of ≥3 social vulnerability criteria (Table 4). Furthermore, among socially disadvantaged but ineligible women, the proportion experiencing social isolation during their pregnancy increased from 15.0% pre-pandemic to 21.5% post-resumption, and the proportion reporting precarious housing rose from 11.7% to 19.8%. Likewise, housing instability worsened across these periods among socially vulnerable women meeting exclusion criteria: the proportion who had moved several times in the previous 12 months increased from 16.6% pre-pandemic to 25.3% post-resumption, and the proportion living in emergency accommodation, portable dwellings, or who were experiencing homelessness increased from 7.1% to 15.4%.

**Table 4:** Distribution of inclusion and exclusion criteria among ineligible women facing social disadvantage, by study period. The ECAIL trial, Lille study area.

	March 2017 to Apr 2023 % (n = 746)	March 2017 to March 2020 % (n = 453)	Sept 2020 to Apr 2023 % (n = 293)	*p*-value
**Inclusion criteria: social vulnerabilities**				
Receives social or medical benefits	75.1	77.0	72.0	0.12
Household's main source of income is not employment	65.1	65.3	64.8	0.89
Perceived financial hardship	31.0	31.1	30.7	0.91
Social isolation	17.6	15.0	21.5	0.02
Precarious housing	14.9	11.7	19.8	0.002
≥ 3 social vulnerabilities	27.1	25.8	29.0	0.34
**Exclusion criteria**				
Planning to move outside the study area before the child's first birthday	30.0	34.4	23.2	0.001
Moved several times in the past 12 months	20.0	16.6	25.3	0.004
Sheltered in emergency centers, portable dwellings or homeless	10.3	7.1	15.4	<0.001
Suboptimal understanding/speaking of French	20.6	17.9	24.9	0.02
Serious illness during pregnancy	18.2	17.9	18.8	0.76
Under legal guardianship	5.0	3.8	6.8	0.059
Multiple pregnancy (≥3 fetuses)	1.1	1.5	0.3	0.16
Illegal substance use (excluding cannabis)	2.4	1.8	3.4	0.15

*p*-values compare the pre-pandemic (March 2017–March 2020) and post-resumption (September 2020–April 2023) periods using χ² tests.

#### Follow-up.

From March 2017 to April 2023, only 2% (n = 46) of the 2,316 expected follow-up visits were skipped, with no significant difference between pre-pandemic and post-resumption periods (Table 2). The rate of failed visits (the dietitian made the trip but the participant was not present) was, however, lower after the study resumed (1.7%) than before the COVID-19 outbreak (4.9%).

### Findings from the qualitative component

The focus group, which lasted 81 minutes, explored how COVID-19 affected home visit delivery, strategies to maintain relationships with participating families, the pivotal moment of the resumption of home visits, and the acceptability of the adapted safety protocol for both dietitians and families. Table 5 presents the key themes and illustrative quotations. During the first lockdown, dietitians expressed uncertainty and concern about whether, when, and how the study would resume. Despite these obstacles, dietitians remained committed to maintaining the study’s home-visit model, considering that remote alternatives, such as telephone or video counseling, were not compatible with the face-to-face nature of the study, which relied on interactions with both parents and children and the delivery of tailored nutritional counselling. Their input was instrumental in designing the subsequent adaptations, as they took part in discussions surrounding implementation under new constraints. Nevertheless, they maintained contact with participating families through telephone calls and newsletters while preparing for the resumption of home visits. These adaptations appeared to have strengthened participant engagement and commitment when home visits became possible again. Both dietitians and participants (as reported by dietitians) expressed gladness at the resumption of the trial. The ECAIL study was authorized to resume before many other clinical studies at Lille University Hospital because of the social vulnerability of its participants. Dietitians reported that mothers described these visits as helping to alleviate social isolation, which had intensified during successive lockdowns. Dietitians did not perceive mask-wearing during hospital screenings or home visits as a barrier to women’s participation or to building rapport during follow-up visits, given the widespread use of masks at the time. Yet they allowed themselves to relax mask use during home visits, with mothers’ authorization, in cases where it hindered interactions with the child. Finally, the pre-visit COVID-19 risk assessment questionnaire was perceived as an effective implementation adaptation, helping to avoid unnecessary trips when participants were unavailable and thereby saving time.

**Table 5:** Qualitative themes retrieved from the focus group and mapped onto RE-AIM dimensions, with illustrative quotations.

RE-AIM dimension	Themes	Sub-themes	Illustrative quotations
Implementation	Theme 1. Maintaining contact with participants while visits were suspended	Proactive relational continuity	*“We stopped but we still kept contact, in any case I kept the link by phone with the ladies, especially when there were the visits that arrived, those of the three months, during that period, in any case I called all the ladies to explain to them that for the moment the ECAIL visits were stopped and that we would keep them informed, we even made a little note … the little newsletter with a little message to encourage them with our photos, it was still important to keep this link even if we didn’t really know what to say to them, but still to say that we were there and that they could contact us if necessary, that we were available by phone.”* – Dietitian 1
Implementation		Maintaining link facilitated commitment	*“That was good, and after, contacting them again to restart the visits, adherence immediate.”* – Dietitian 1
Adoption	Theme 2. Resuming home visits	Uncertainty regarding the resumption of home visits	*“In any case, for me, it was a fear of telling myself how we were going to return to people afterward? Can we go back? Because it was so unclear at the beginning, everything was at a standstill … And therefore to tell ourselves how we are going to find the ladies? Do we leave them like this? […] Will we be able to resume? And if we resume will it always be, will it be the same?”* – Dietitian 1
Reach		Participants’ happiness at the resumption of home visits	*“The ladies were glad to see people again, and so, I remember that the schedule was full of visits because they were glad … the ladies were really, really happy to have someone come to the house because it was still the lockdown, so someone coming to the house to chat … glad to have a social link with someone from outside again, not having their family locked inside their apartment.”* – Dietitian 2
Adoption		Dietitians’ engagement	*“I was also glad to resume the visits.”* – Dietitian 2
Implementation		Necessity of maintaining home-visits as a core feature of the intervention	“For us, with ECAIL visits, video calls aren’t even an option; we can’t do the visit via video (Dietictian 1). Not even phone calls? (DP). No (Dietitian 5). We can’t do a visit over the phone; it’s not possible. All the tools that were put in place as we went along just didn’t suit us (Dietitian 1)”
Implementation		Longer visits after resumption	*“However, clearly, the visits, at least in my recollection, I'm not sure if it's biased by the fact that it's dated, but the visits lasted a long time.”* – Dietitian 2
Adoption		Prioritization of the ECAIL study resumption among other trials	*“And many protocols for healthy volunteers didn't resume, didn't resume at that time. They made a real exception for the ECAIL study because of its specific audience, and we measured that there were more benefits in going back to people than waiting further to avoid the risk of contamination.”* – Dietitian 5
Implementation	Theme 3. Adaptation of protocol	Safety protocol as a condition for visit resumption	*“It's true that we were allowed to resume visits in June only if we implemented this safety protocol... we had to implement this protocol to protect both the families and the dietitians.”* – Dietitian 5
		Acceptability of masks for participants	*“We saw the ladies as part of the continuation of their prenatal consultation … it was something from everyday life [the mask] it was mandatory in public places, in public transportation … so it became customary, actually. So even more at the hospital, the place where it is necessary to wear the mask. So, as a result, I think that it was logical, well, it was the most logical place, and I don't think that it posed a problem for them, that it was a barrier.”* – Dietitian 1
			“*In fact, it was an everyday thing, the mask eventually became part of daily life, so it wasn't something that presented a barrier.*” – Dietitian 1
		Acceptability of masks for children	“*I can see that the mask is a barrier for the children, so I take it off in those situations; if I see that it’s too difficult for the child to see me with a mask on, I ask the lady if that’s possible*” – Dietician 6
		Pre-visit COVID-19 risk assessment questionnaire: reduction of missed visits and time-saving	“*It's true that we had this reflection, well, we arrived directly with the COVID questionnaire, there were many fewer trips with no one home, for nothing*.” – Dietitian 3
			“*So, this allowed for efficiency, and, well, it had unforeseen positive consequences, in the sense that there were fewer unnecessary trips for nothing, less time wasted*.” – Dietitian 5

## Discussion

This study of an interdisciplinary public health intervention among socially disadvantaged families provides implementation-relevant insights into how trial implementation evolved across the COVID-19 pandemic and the subsequent cost-of-living crisis. Interpreted through a RE-AIM lens [19,20], our findings suggest that these contextual shocks were associated with an increase in the proportion of women meeting the trial eligibility criteria, without compromising provider *Adoption* or *Implementation*, and may even have reinforced engagement during key phases of delivery. Quantitative and qualitative findings converged, showing shifts in eligibility and participation profiles alongside sustained involvement of both implementers and participants. This resulted in a stable rhythm of home visits after resumption, with some parents eager to re-establish social contact, suggesting that home visits may have provided benefits extending beyond intervention delivery. These health and economic shocks also affected socially disadvantaged families not eligible for the ECAIL trial, exacerbating existing social and material vulnerabilities. Taken together, these findings suggest overall robust implementation despite major contextual disruptions and may inform the design of more resilient interventions.

### Eligibility, ineligibility, and social vulnerabilities

The consistently high eligibility rate observed in the ECAIL trial, even before the pandemic, is consistent with the well-documented socioeconomic disadvantage of the Hauts-de-France region, shaped by the economic decline following the collapse of its coal, textile, and steel industries in the 1980s [30]. In 2020, 17.2% of the population lived below the poverty line, compared with 13.6% nationally [24,31]. The *Nord* district, where Lille is located, also has the highest poverty rate in the region (18.4%) [24], together with higher levels of unemployment [32], lower educational attainment [33,34], a greater proportion of blue-collar workers [33,35], and more single-parent families [36,37].

Against this already disadvantaged socioeconomic backdrop, the higher post-resumption eligibility rate observed in ECAIL suggests that the COVID-19 pandemic further exacerbated existing inequalities. This interpretation is consistent with national evidence showing that low-income households were disproportionately affected by labor-market disruptions and were less likely to hold stable jobs compatible with teleworking [38]. As a result, household living standards declined. Labor-market disruptions, including those affecting lower middle-class households, likely contributed to the higher post-resumption eligibility rate, consistent with the increased proportion of women reporting that employment was not their household’s main source of income during P1. Likewise, at the national level, the poverty rate increased from 13.6% in 2020 to 15.4% in 2023 [31].

The higher proportion of ECAIL-eligible women receiving social or medical benefits during P1 is consistent with national administrative data showing a sharp increase in benefit receipt early in the pandemic [39]. This may help explain the concurrent decrease in perceived financial difficulties. Temporary reductions in household spending during lockdowns, when consumption expenditure fell markedly [40], may also have played a role in reducing short-term financial pressures for some households.

The persistently high eligibility rate nonetheless observed during P2 and P3 suggests that the socioeconomic consequences of the pandemic extended well beyond the acute health crisis, likely reflecting persistent macro-economic pressures, including rising energy and food prices. Although employment partially recovered after restrictions were lifted, inflation and the withdrawal of exceptional government financial support introduced during the pandemic likely contributed to the renewed increase in reported financial difficulties during P3. More broadly, the cost-of-living crisis compounded the challenges faced by a population already weakened by the pandemic. This interpretation is consistent with national surveys documenting worsening financial hardship and food insecurity during this period [41].

This worsening socioeconomic context is also reflected in our analysis of women ineligible for the ECAIL trial despite experiencing social vulnerability. These women faced increasing housing precarity and instability, as well as heightened social isolation, consistent with previous findings from the Haut-de-France region [42]. These findings also suggest that the minimum level of stability required to participate in longitudinal intervention studies may inadvertently exclude some women experiencing social vulnerability, an issue that warrants further investigation. Future research should explore strategies to reduce participation burden and develop implementation approaches that better reach families experiencing the highest levels of social disadvantage.

Taken together, these results underscore how multidimensional crises such as the COVID-19 pandemic can intensify pre-existing vulnerabilities, highlighting the need for resilient intervention designs.

### Participation

Participation in the ECAIL trial increased slightly during the post-resumption P1 and P2 subperiods. From an *Implementation* perspective, these findings are consistent with the dieticians’ perception that protective measures, including mask wearing, did not substantially interfere with screening interviews. From an *Adoption* perspective, this was likely associated with a higher frequency of face-to-face delivery of study information at the maternity ward, rather than telephone contact. This shift may have resulted from the prioritization of the ECAIL trial after study resumption, which likely created favorable conditions for implementation. As highlighted in the qualitative findings, ECAIL was authorized to resume before many other clinical studies because of the social vulnerability of its participants, illustrating an equity-oriented approach. Direct interactions between dietitians and women (and their partners, when present) may have facilitated participant recruitment more effectively than delayed, impersonal phone calls, although this depended on HCPs contacting the dietitians during prenatal appointments. In this context, regular meetings between the ECAIL team and HCPs likely supported sustained and synergistic collaboration despite pandemic-related pressures on hospital services. Together, quantitative and qualitative findings support the interpretation that coordination between dietitians and HCPs was strengthened during the early post-resumption period (P1). However, HCP engagement appeared to decline slightly during period P3, in parallel with the decrease in participation. This may reflect increased workload following the resumption of other clinical trials, compounded by persistent disruptions and time constraints affecting hospital services. The slight decrease in participation during P3 may also reflect the cumulative burden of successive crises, which likely reduced women’s availability and willingness to participate, as suggested by the more frequent reporting of time constraints and unavailability among those who declined participation post-resumption. These findings highlight the prolonged and evolving impact of crises, underscoring the importance of considering temporal dynamics in their effects.

### Follow-up

Across both qualitative and quantitative components, findings converged in suggesting that dietitians and families were eager for the resumption of home visits. During the first lockdown, dietitians maintained regular contact with participants. This continuity of contact emerged as a key implementation mechanism for sustaining family engagement, as reflected in the very low rate of skipped home visits. The implementation of the COVID-19 safety protocol facilitated pre-visit exchanges, reduced potential apprehension among participants, and contributed to fewer missed appointments.

These observations have significant methodological implications and highlight the value of ongoing relational work between follow-up visits. This requires additional human resources and research time, particularly when working with socially disadvantaged populations, who are known to be “hard-to-survey” [21,22]. However, this investment may improve the efficiency of trial implementation by reducing unnecessary trips and facilitating visit rescheduling, thus saving time and subsequent manhour costs in the long term, while also enhancing adherence to the study protocol, participant commitment, and data quality by limiting missed visits. More broadly, our findings reinforce the relevance of home-based delivery for reaching and engaging populations facing social adversity, who often have lower utilization of health care and community services and experience greater social isolation [43,44], while facilitating one-on-one interactions that foster trust between providers and mothers and enable more personalized and context-sensitive interventions [18]. This may partly explain why mothers perceived the resumption of home visits as particularly valuable after successive lockdowns, when opportunities for social interaction had been substantially reduced.

### Perspectives

This study examined how the distribution of social vulnerability criteria differed between eligible and ineligible women, as well as between participants and nonparticipants, an aspect that remains rarely explored in implementation research [44]. It also illustrates how such data can be collected and analyzed to evaluate intervention reach [45]. These findings provide important insights for interpreting the trial’s external validity and the potential generalizability of its future effectiveness findings. Additionally, qualitative interviews are currently being conducted with participating mothers to explore their motivations, contextual constraints, and perceptions of participation. These complementary data will contribute to a more comprehensive process evaluation of this complex public health intervention [46–49].

### Limitations and strengths

One limitation of this study is that the qualitative component relied exclusively on a focus group with dietitians, whose perspectives may not fully reflect those of other stakeholders involved in trial implementation. Participation in the focus group was also unequal, with two dietitians contributing more extensively than the others, although quotations were selected to illustrate the identified themes rather than to reflect the relative frequency of individual contributions. Because the focus group was conducted two years after the last lockdown, recall bias cannot be excluded. Moreover, the before-and-after observational design without a control group precludes attributing the observed changes specifically to the COVID-19 pandemic and the cost-of-living crisis, as other temporal influences cannot be excluded. Finally, because the quantitative analyses were restricted to the Lille site, where recruitment started before the pandemic, the findings may not be fully generalizable to other implementation settings. Nonetheless, the vividness of the dietitians’ recollections underscores the lasting impact of this period and the relevance of capturing these experiences retrospectively. Although effectiveness outcomes are not examined here, limiting conclusions about whether successful implementation translated into intervention benefit, this focus is consistent with the RE-AIM framework’s emphasis on disentangling *reach*, *adoption*, and *implementation* processes as necessary precursors to effectiveness and sustainability [19]. A major strength lies in the triangulation of quantitative and qualitative methods [28,44,47], which enabled a richer understanding of both the evolving characteristics of the target population and the implementation processes of the intervention across successive health and economic crises. Importantly, the inclusion of dietitians’ perspectives represents a further strength because they were the frontline implementers of the trial and directly involved in participant recruitment, follow-up and data collection. Their perspectives, which are still relatively rarely captured in implementation research, provided valuable insights into day-to-day implementation during the successive crises. The study’s focus on pregnant women experiencing social vulnerabilities, a heterogeneous population that remains largely underrepresented in epidemiological research, provides new knowledge about the specific barriers to intervention delivery and participant engagement in a context of worsening poverty. The adjustments made after the onset of the COVID-19 pandemic illustrate the flexibility of this complex interventional research in responding to unforeseen events and adapting implementation to enable study continuation [46,48]. They also suggest that home visits and one-to-one interactions may strengthen participation and commitment, despite constraints on face-to-face contact.

## Conclusion

The social impact of both the pandemic and the cost-of-living crisis, often described as the “second health emergency” [5], was associated with a higher eligibility rate among socially disadvantaged families in the ECAIL trial, while participation remained stable. These findings provide important insights into how successive crises may influence participation in programs designed to mitigate social disadvantage. In the ECAIL trial, the collaborative foundations of this co-created intervention may have helped sustain both stakeholder and participant engagement despite increased workload and organizational strain experienced within hospitals during the successive crises. Moreover, home visits and telephone calls between follow-ups were perceived as effective implementation strategies, not only for reaching families and supporting adherence, but also for mitigating social isolation among participants. Overall, these findings suggest that interventions co-designed with stakeholders and target populations, and supported by appropriate implementation strategies, may remain broadly resilient during major external disruptions, highlighting the value of flexible, context-sensitive implementation approaches in a context of rising poverty and ongoing geopolitical and economic instability.

## Supporting information

S1 File Eligibility questionnaire co-designed with healthcare providers from the maternity ward of the Lille University Hospital to assess women's eligibility for the ECAIL trial. (PDF)

S2 File Strobe. (DOCX)

## Abbreviations

ECAIL: prEgnanCy and eArly Childhood nutrItion triaL
HCP: healthcare provider
Insee: National Institute of Statistics and Economic Studies
Inserm: National institute for health and medical research
RE-AIM: Reach, Effectiveness, Adoption, Implementation and Maintenance
