# Supplementary material for: Reach and engagement in the ECAIL trial targeting socially disadvantaged families across the COVID-19 pandemic and cost-of-living crisis: A mixed-methods implementation study
Source: PLoS One. 2026 Oct 8;21(10):e0350372. doi: 10.1371/journal.pone.0350372 (PMC13649008; doi:10.1371/journal.pone.0350372)
Supplement: Supplementary file 1 [file pone.0350372.s001.pdf]

Reach and engagement in the ECAIL trial targeting socially disadvantaged families across the COVID-19 pandemic and cost-of-living crisis: a mixed-methods implementation study.

Supplementary document 1. Eligibility questionnaire co-designed with healthcare providers from the maternity ward of the Lille University Hospital to assess women's eligibility for the ECAIL trial

Front of the questionnaire:

CHU LILLE

Jeanne de Flandre Maternity Hospital
Prenatal consultations
Living conditions questionnaire

Please complete this questionnaire in the waiting room.
- Staff in the waiting room can help you fill it in if necessary -

Under certain conditions, the Jeanne de Flandre Maternity Hospital currently offers nutritional support, from pregnancy until your child is 24 months old.

1. Is this your 1st **prenatal visit** to the maternity hospital for the current pregnancy?	☐ Yes *EXCL.*	☐ No
2. **Do you live in one of the following cities?** ☐ Lille ☐ Faches-Thumesnil ☐ Haubourdin ☐ Hellemmes ☐ La Madeleine ☐ Lambersart ☐ Lomme ☐ Loos ☐ Mons-en-Barœul ☐ Marcq-en-Barœul ☐ Ronchin ☐ St-André-Lez-Lille ☐ Villeneuve-d'Ascq ☐ Wasquehal ☐ Wattignies	☐ Other, specify: ------------	
3. Do you intend to **move** to a city **other than those listed above** before your baby is 12 months old?	☐ Yes	☐ No
4. Are you a **minor**? (under 18 years old)	☐ Yes	☐ No
5. Are you under **guardianship**?	☐ Yes	☐ No
6. Are you pregnant with **triplets (or more)**?	☐ Yes	☐ No
7. Have you **moved house** <u>several times</u> in the last 12 months?	☐ Yes	☐ No
8. Do you live in an emergency shelter (or a hostel accessible through 115), a caravan, a mobile home, a trailer or other mobile dwelling, or in a place not intended for habitation (street, vehicle, building lobby, makeshift shelter)?	☐ Yes	☐ No
9. In the last 12 months, have you ever used **any of these substances:** ecstasy, amphetamines, crack, LSD, cocaine, heroin?	☐ Yes	☐ No
(to be confirmed by the healthcare provider (on the back)) 10. Are you currently being treated for a **chronic illness**? ○ Heart disease, respiratory failure, **type 1 diabetes** ○ Liver disease (cholestasis), kidney failure, immune deficiency ○ Intestinal malabsorption syndrome (cystic fibrosis, intestinal resection, etc.) ○ Hereditary metabolic disease (enzymatic) ○ Known chronic neurological or psychiatric condition ○ Other serious or progressive chronic illness (advanced cancer), please specify:	☐ Yes	☐ No
	INCL.	
11. Do you receive **any of these benefits: CSS** (Complementary Health Solidarity), which corresponds to the former **CMU-c** (Complementary Universal Health Coverage) or **ACS** (assistance with the payment of complementary health insurance), **AME** (State Medical Assistance), **RSA** (Active Solidarity Income), the **Activity Bonus, AAH** (Disabled Adult Allowance), or **ASS** (Specific Solidarity Allowance)?	☐ Yes	☐ No
12. Do you have periods during the month where you experience **significant financial difficulties** in meeting your needs (food, medication, rent, bills, etc.)?	☐ Yes	☐ No
13. Will you be **taking care of your baby alone after birth** (that is, <u>without the presence</u> of a partner or another adult to help you)?	☐ Yes	☐ No
14. What is your **main source of income** (for you and your partner)? a. Work ☐ b. Family/friends ☐ c. Social assistance ☐ d. None ☐		
15. Is your **accomodation** ?		
- a hostel room (paid for by you or your partner)	☐ Yes	☐ No
- a social hostel room	☐ Yes	☐ No
- social rehabilitation centre (CHRS)	☐ Yes	☐ No
16. Is any of the following **facilities missing** from your housing: shower (or bathtub), toilet, running water, or heating system?	☐ Yes	☐ No

17. What is your telephone number? __ __ .__ __ .__ __ .__ __ .__ __

Date : |__|__|/ |__|__|/ |__|__|

Thank you for completing this questionnaire ☺

➥ **Questionnaire to be given to the healthcare provider or dietitian**

Questionnaire Bleu, v3_05.07.2021 (traduit en anglais)

Back of the questionnaire:

Section strictly reserved for healthcare providers

- **Patient understands and speaks French:** ☐ **YES** ☐ no
- **Disease(s)**, as indicated on the front (Q10), **preventing the patient from being included in ECAIL** ⓘ ☐ yes ☐ **NO**
- **Patient participating in another randomized clinical trial incompatible with ECAIL (ePPOP)** ☐ yes ☐ **NO**

ⓘ Disease = exclusion criterion if:
-Likely to disrupt the child's growth and cognitive development;
-Incompatible with regular participation in the study.

ⓘ *Note: Type 2 diabetes is not a criterion for exclusion*

►If yes, which trial: ....

ECAIL +	ECAIL -
☐	☐
►TSI dietitians at 83555	► Box

Name of the healthcare provider: ....

Section reserved for dietitians

Detailed information about the study

- **Detailed information about the study conducted:**

☐ in person ☐ by telephone date: |__ | __| |__ | __| |__ | __| ☐ No

- **Information letter given to the patient:**

☐ in person ☐ by mail date: |__ | __| |__ | __| |__ | __| ☐ No

Name of the dietitian: ....

Phone contact after detailed information about the study

DATE: |__ | __| |__ | __| |__ | __|

Patient agrees to participate in the study: ☐ YES ☐ NO

- ***If no**, reasons for refusal:*
  1) ....
  2) ....
  3) ....
- ***If yes**, V2 visit scheduled for:* |__ | __| |__ | __| |__ | __|

Name of the dietitian: ....

☐ Blue questionnaire completed and validated on: |_ | _| |_ | _| |_ | _|
