# Supplementary material for: Reach and engagement in the ECAIL trial targeting socially disadvantaged families across the COVID-19 pandemic and cost-of-living crisis: A mixed-methods implementation study
Source: PLoS One. 2026 Oct 8;21(10):e0350372. doi: 10.1371/journal.pone.0350372 (PMC13649008; doi:10.1371/journal.pone.0350372)
Supplement: Supplementary file 2 [file pone.0350372.s004.pdf]

Paris, July 17, 2026

To the Editors-in-Chief of *PLOS ONE*

Dear Editors,

Please find enclosed the revised version of our manuscript, entitled "Reach and engagement in the ECAIL trial targeting socially disadvantaged families across the COVID-19 pandemic and cost-of-living crisis: a mixed-methods implementation study" (Manuscript Number: PONE-D-26-23207).

The authors are grateful for the opportunity to revise the manuscript and for the valuable comments and suggestions provided by the reviewers. We have carefully considered all comments and revised the manuscript accordingly. Changes have been highlighted in yellow throughout the tracked-changes version of the manuscript.

Please also find enclosed our point-by-point response to the reviewers' comments, in which changes are highlighted in yellow. All line numbers refer to the revised version of the manuscript (without tracked changes).

We hope that our modifications adequately address the Reviewers' comments and that our manuscript is now suitable for publication in *PLOS ONE.*

Yours sincerely,

Sandrine LIORET, on behalf of all co-authors
OPPaLE (Obstetric, Perinatal, Paediatric, Lifecourse Epidemiology) team, Université Paris Cité and Université Sorbonne Paris Nord, Inserm, INRAE, Centre for Research in Epidemiology and Statistics, Paris, France [**sandrine.lioret@inserm.fr]**

Review Comments to the Author

Please note that funding information should not appear in any section or other areas of your manuscript. We will only publish funding information present in the Funding Statement section of the online submission form. Please remove any funding-related text from the manuscript.

We have removed the funding-related text from the manuscript accordingly.

Please include your full ethics statement in the 'Methods' section of your manuscript file. In your statement, please include the full name of the IRB or ethics committee who approved or waived your study, as well as whether or not you obtained informed written or verbal consent. If consent was waived for your study, please include this information in your statement as well.

We have revised the ethics statement by adding the word "informed", as requested, and by including the full name of the ethics committee, as follows (pages 10-11, lines 238-243):

> The ECAIL RCT has approval from the committee for protection of persons engaged in research (*Comité de Protection des Personnes Nord-Ouest 1,* CPP, 24/07/2014), the national advisory committee on information processing in health research (*Comité consultatif sur le traitement de l'information en matière de recherche,* CCTIRS, 04/09/2014), the national data protection authority (*Commission nationale de l'informatique et des libertés*, CNIL, 15/06/2016). The protocol for the qualitative component was approved by the CPP on 28/06/2021. Written informed consent was obtained from all participants.

Information on written informed consent was already included in the original manuscript, as follows:

> Page 7, lines 152-153: During the baseline home visit, the mother-to-be provided written informed consent for the participation of the mother-child dyad, randomization was performed, and the inclusion process was formalized.
>
> Page 10, lines 225-226: Written informed consent was obtained from each dietitian.

Reviewer #1:

1.1. One of the best-written introductions I've ever reviewed, but the rest of the submission is poorly organized and I'm not sure whether the study is sound because it loses its cohesion. If this study is mixed methods, the methods must inform one another, demonstrate how the quantitative data affects the qualitative analysis (or vice versa - ideally both) and there must be some sort of plan or citation (ideally both) for how to go about developing interconnected analysis. What is here at present is a lot of descriptive statistics without significance, followed by a rushed qualitative report (I hesitate to call it thematic since there is no theory behind how themes are identified or reported) on a focus group where 2/6 participants deliver 90% of the quotations.

We thank the reviewer for this thoughtful comment. We agree that the mixed-methods design and its underlying rationale were not sufficiently explicit in the original manuscript. Our intention was not to use one component to inform the design or analysis of the other, as in sequential mixed-methods designs, but rather to adopt a convergent mixed-methods approach. Quantitative and qualitative data were collected and analyzed independently, and their findings were subsequently triangulated to provide complementary perspectives on implementation processes and strengthen the interpretation of the results.

To clarify this rationale, we have revised the Title, Abstract, Introduction, and Methods sections. We now explicitly describe the study as a convergent mixed-methods design and clarify that the qualitative

component was intended to complement and contextualize the quantitative implementation indicators through triangulation, rather than to explain them sequentially.

> Title: Reach and engagement in the ECAIL trial targeting socially disadvantaged families across the COVID-19 pandemic and cost-of-living crisis: a mixed-methods implementation study
>
> Abstract (page 2, lines 35-37): **Methods:** Using a convergent mixed-methods design, we analyzed 5,744 eligibility questionnaires distributed at the maternity ward and conducted a focus group with six dieticians delivering the intervention to triangulate quantitative implementation indicators with qualitative implementation data.
>
> Introduction (page 4, lines 93-96): Building on an interdisciplinary implementation science approach, this study adopts a convergent mixed-methods design to triangulate quantitative and qualitative findings and provide complementary insights into trial implementation (25).
>
> Methods:
> Page 8, lines 171-176: **Overall mixed-method design**
> This study adopted a convergent mixed-methods design (25). Quantitative and qualitative data were collected and analyzed independently to examine complementary dimensions of implementation. Integration occurred during interpretation through triangulation of findings, with the aim of assessing the convergence, complementarity, or divergence between implementation indicators derived from routine trial data and dietitians' experiences of intervention delivery.
>
> Page 10, lines 227-229: In line with the convergent mixed-methods design (25), the qualitative component was intended to complement and contextualize the quantitative implementation indicators through triangulation, rather than explain them sequentially.

We have also added a methodological reference describing the principles of convergent mixed-methods designs and data integration:

> Reference n°25: Fetters, M.D., Curry, L.A. and Creswell, J.W. (2013), Achieving Integration in Mixed Methods Designs—Principles and Practices. Health Serv Res, 48: 2134-2156. https://doi.org/10.1111/1475-6773.12117

Regarding the statistical analysis, our primary objective was to describe implementation outcomes (e.g., reach, participation, adoption) before and after the COVID-19 disruption rather than to test causal hypotheses. Consequently, several implementation indicators are presented descriptively, as is common in implementation research, while formal statistical comparisons were conducted whenever appropriate. We have clarified this objective in the Methods section, as follows (page 11, lines 247-250):

> The quantitative analysis primarily consisted of describing implementation-related indicators across study periods, in line with the RE-AIM framework. Where appropriate, formal statistical comparisons were performed to assess differences either between the two main study periods (pre-pandemic vs post-study resumption) or across the four study periods (P0–P3), depending on the analysis presented.

Regarding the qualitative analysis, we agree that its description lacked detail. We have expanded the Methods section to better describe the thematic content analysis, including how themes were developed, reviewed, and refined, as well as the role of independent review in the coding process, (pages 11-12, lines 264-270):

> DP manually transcribed the focus group verbatim and imported it into NVivo software. A thematic content analysis (29) was then conducted. The transcript was coded by DP, and codes were iteratively grouped into broader themes reflecting implementation context, disruptions, adaptations, and perceived mechanisms influencing reach and engagement. The coding and resulting themes were independently reviewed by PS, and any discrepancies were discussed until consensus was reached. Illustrative quotations were then selected to exemplify the identified themes and subthemes and reflect dietitians' shared experiences of trial implementation during the pandemic.

We acknowledge that participation in the focus group was unequal, with two participants contributing more extensively to the discussion than the others, as is often the case in focus groups. However, our intention

was not to quantify individual contributions but to identify and illustrate the themes emerging from the group discussion. Accordingly, quotations were selected for their ability to exemplify the identified themes rather than to reflect the relative frequency of individual contributions. We have nevertheless acknowledged the unequal distribution of contributions as a limitation in the revised manuscript (pages 24-25, lines 519-521).

> Participation in the focus group was also unequal, with two dietitians contributing more extensively than the others, although quotations were selected to illustrate the identified themes rather than to reflect the relative frequency of individual contributions.

1.2. One of the fundamental flaws is that while multiple time periods are introduced and compared, no information is given about how these time periods might vary in the healthcare setting. There are brief mentions of multiple lockdowns, masking standards, and relaxation of mitigations, but we never see these laid out, let alone conferred with the timeline of data collection and patient engagement. What conventions changed over these four periods? Don't assume that COVID was the same for all readers - walk them through what it was like in Lille. Contextualize it with brief comparisons to other cities, the rest of France and/or Europe, the rest of the world.

The study timeline was already presented in Figure 1, including the timing of the successive national lockdowns, the suspension and resumption of the trial, and the duration of the COVID-19 safety protocol. However, we agree that the relationship between these successive implementation phases and the evolving healthcare context was not sufficiently explicit in the text. We have therefore revised the Methods section to clarify both the rationale for dividing the post-resumption period into three approximately one-year periods, in order to capture successive implementation phases while ensuring comparable seasonal coverage across periods, and the implementation context associated with each phase. In particular, we now explain that these periods corresponded to successive changes in national public health restrictions and hospital infection-control procedures (including mandatory masking and the ECAIL-specific COVID-19 safety protocol), and we refer more explicitly to Figure 1 when describing the study timeline, as follows (page 8, lines 180-189):

> We defined two periods, namely pre-pandemic (March 2017 to March 2020; P0) and post-study resumption (September 2020 through April 2023). As shown in **Fig 1**, the post-resumption period encompassed successive phases of the COVID-19 pandemic and the progressive relaxation of public health measures. These phases corresponded to successive changes in national public health restrictions and hospital infection-control procedures that characterized the implementation context of the ECAIL trial, including mandatory masking and the study-specific COVID-19 safety protocol introduced upon trial resumption. To capture temporal variation in implementation conditions, the post-resumption period was segmented into three approximately one-year periods (September-August). This segmentation also ensured comparable seasonal coverage across periods. The final period was shorter due to the end of screening in April 2023 (**Fig 1**; P1, P2, and P3).

In Figure 1, we have also replaced “COVID-19 health protocol” with “COVID-19 safety protocol implemented upon trial resumption”, as follows:

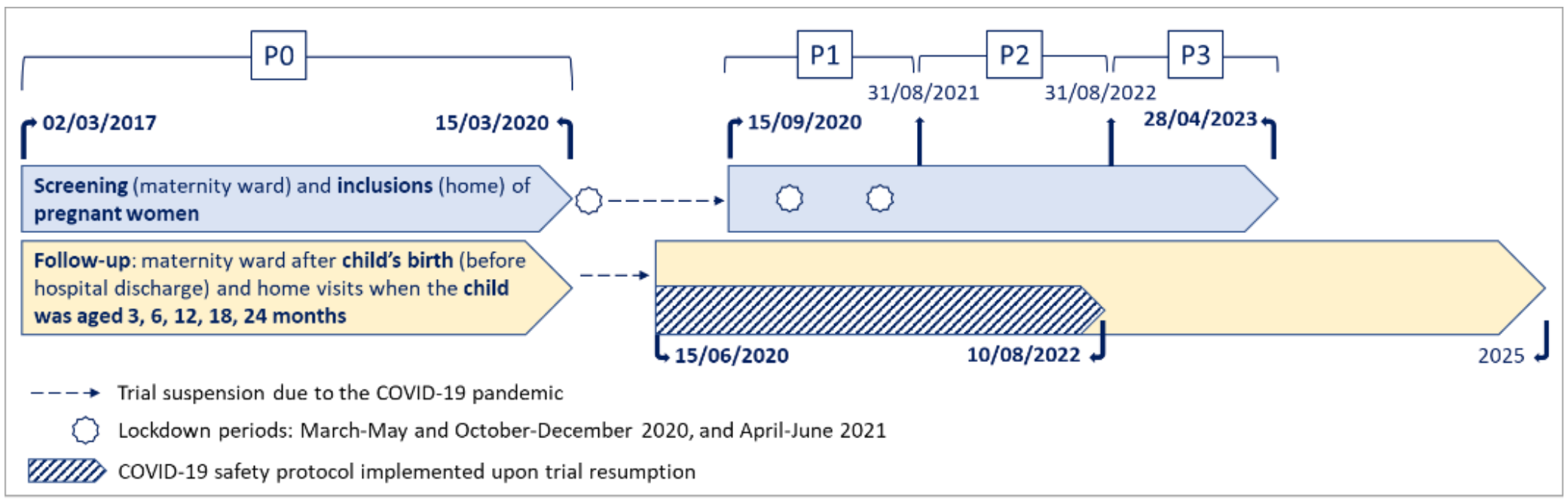

1.3. Discussion section introduces far too many new topics to an already-dense study; authors shouldn't have to cite much by this point, it should all be part of the introduction, methods, and data sections, then

outlined in analysis and reported in results, and then under discussion nothing is left except to reflect on implications of findings and future directions.

We agree that the Discussion should primarily focus on interpreting the study findings and their implications. Our intention was to interpret our findings in light of the existing literature rather than introduce new concepts or hypotheses. Nevertheless, we have revised the subsection entitled "**Eligibility, ineligibility, and social vulnerabilities**" to improve its focus by streamlining some contextual background and strengthening the links between our findings and the supporting literature. More specifically, we restructured the subsection so that each paragraph now begins with the study findings, followed by their interpretation and supporting evidence from the literature, as illustrated below (pages 21-22, lines 420-459):

Eligibility, ineligibility, and social vulnerabilities

The consistently high eligibility rate observed in the ECAIL trial, even before the pandemic, is consistent with the well-documented socioeconomic disadvantage of the Hauts-de-France region, shaped by the economic decline following the collapse of its coal, textile, and steel industries in the 1980s (30). In 2020, 17.2% of the population lived below the poverty line, compared with 13.6% nationally (24, 31). The *Nord* district, where Lille is located, also has the highest poverty rate in the region (18.4%) (24), together with higher levels of unemployment (32), lower educational attainment (33, 34), a greater proportion of blue-collar workers (33, 35), and more single-parent families (36, 37).

Against this already disadvantaged socioeconomic backdrop, the higher post-resumption eligibility rate observed in ECAIL suggests that the COVID-19 pandemic further exacerbated existing inequalities. This interpretation is consistent with national evidence showing that low-income households were disproportionately affected by labor-market disruptions and were less likely to hold stable jobs compatible with teleworking (38). As a result, household living standards declined. Labor-market disruptions, including those affecting lower middle-class households, likely contributed to the higher post-resumption eligibility rate, consistent with the increased proportion of women reporting that employment was not their household's main source of income during P1. Likewise, at the national level, the poverty rate increased from 13.6% in 2020 to 15.4% in 2023 (31).

The higher proportion of ECAIL-eligible women receiving social or medical benefits during P1 is consistent with national administrative data showing a sharp increase in benefit receipt early in the pandemic (39). This may help explain the concurrent decrease in perceived financial difficulties. Temporary reductions in household spending during lockdowns, when consumption expenditure fell markedly (40), may also have played a role in reducing short-term financial pressures for some households.

The persistently high eligibility rate nonetheless observed during P2 and P3 suggests that the socioeconomic consequences of the pandemic extended well beyond the acute health crisis, likely reflecting persistent macro-economic pressures, including rising energy and food prices. Although employment partially recovered after restrictions were lifted, inflation and the withdrawal of exceptional government financial support introduced during the pandemic likely contributed to the renewed increase in reported financial difficulties during P3. More broadly, the cost-of-living crisis compounded the challenges faced by a population already weakened by the pandemic. This interpretation is consistent with national surveys documenting worsening financial hardship and food insecurity during this period (41). This worsening socioeconomic context is also reflected in our analysis of women ineligible for the ECAIL trial despite experiencing social vulnerability. These women faced increasing housing precarity and instability, as well as heightened social isolation, consistent with previous findings from the Haut-de-France region (42). These findings also suggest that the minimum level of stability required to participate in longitudinal intervention studies may inadvertently exclude some women experiencing social vulnerability, an issue that warrants further investigation. Future research should explore strategies to reduce participation burden and develop implementation approaches that better reach families experiencing the highest levels of social disadvantage.

Taken together, these results underscore how multidimensional crises such as the COVID-19 pandemic can intensify pre-existing vulnerabilities, highlighting the need for resilient intervention designs.

In addition, several other parts of the Discussion were revised in response to reviewers' comments to improve the distinction between study findings and their interpretation, temper causal language, reduce speculative statements, and strengthen the focus on the implications of the findings, e.g.:

Pages 22-23, lines 461-476: **Participation**

Participation in the ECAIL trial increased slightly during the post-resumption P1 and P2 subperiods. From an *Implementation* perspective, these findings are consistent with the dieticians' perception that protective measures, including mask wearing, did not substantially interfere with screening interviews. From an *Adoption* perspective, this

was likely associated with a higher frequency of face-to-face delivery of study information at the maternity ward, rather than telephone contact. This shift may have resulted from the prioritization of the ECAIL trial after study resumption, which likely created favorable conditions for implementation. As highlighted in the qualitative findings, ECAIL was authorized to resume before many other clinical studies because of the social vulnerability of its participants, illustrating an equity-oriented approach. Direct interactions between dietitians and women (and their partners, when present) may have facilitated participant recruitment more effectively than delayed, impersonal phone calls, although this depended on HCPs contacting the dietitians during prenatal appointments. In this context, regular meetings between the ECAIL team and HCPs likely supported sustained and synergistic collaboration despite pandemic-related pressures on hospital services. Together, quantitative and qualitative findings support the interpretation that coordination between dietitians and HCPs was strengthened during the early post-resumption period (P1).

Page 24, lines 495-504: **Follow-up**

(…) However, this investment may improve the efficiency of trial implementation by reducing unnecessary trips and facilitating visit rescheduling, thus saving time and subsequent manhour costs in the long term, while also enhancing adherence to the study protocol, participant commitment, and data quality by limiting missed visits. More broadly, our findings reinforce the relevance of home-based delivery for reaching and engaging populations facing social adversity, who often have lower utilization of health care and community services and experience greater social isolation (43, 44), while facilitating one-on-one interactions that foster trust between providers and mothers and enable more personalized and context-sensitive interventions (18). This may partly explain why mothers perceived the resumption of home visits as particularly valuable after successive lockdowns, when opportunities for social interaction had been substantially reduced.

1.4. Tables are sloppy, and much of the information shown there could be relegated to supplemental materials. Tables 2, 3, and 4: Please indicate percentage in the headers of relevant columns rather than the table header. I'm not always clear which column these p-values (note the lowercase p) report for. Also, please indicate items where p-value is statistically significant with an asterisk (*). Table 5 is dominated by only Dietitians 1 and 2; some of the quotations are even redundant.

We thank the reviewer for these helpful suggestions. We have revised Tables 2–4 to improve their readability by indicating percentages in the relevant column headers, clarifying in the table footnotes what the reported *p*-values compare, and using a lowercase *p* throughout. We retained the exact *p*-values without adding significance asterisks, as this is consistent with the reporting style adopted by PLOS ONE and provide more information than significance thresholds alone.

Regarding the suggestion to move these tables to the Supplementary Material, we respectfully chose to retain them in the main manuscript, as they present the main quantitative findings of the study. Specifically, they report the implementation outcomes central to our study objectives, including reach, participation, and implementation, and therefore constitute essential results rather than supplementary information.

Finally, regarding Table 5, as is often the case in focus groups, participation was unequal, with two participants contributing more extensively to the discussion than the others. We have acknowledged this as a limitation in the revised manuscript (pages 24-25, lines 519-521). We nevertheless retained the selected quotations because they illustrate distinct aspects of the identified themes rather than the frequency of individual contributions.

Participation in the focus group was also unequal, with two dietitians contributing more extensively than the others, although quotations were selected to illustrate the identified themes rather than to reflect the relative frequency of individual contributions.

1.5. Supplemental documents are attached but not introduced/discussed. Standardized questionnaires can just be introduced and cited, but they must be introduced.

The only Supplementary Document intended for publication is the eligibility questionnaire (Supplementary Document 1), which is introduced in the Methods section when first described, as follows (page 5, lines 123-129):

> While waiting for their prenatal appointment, pregnant women were given an eligibility questionnaire (**Supplementary document 1**) by reception staff, covering all inclusion and exclusion criteria (**Table 1**). Some forms of precarious housing, such as substandard living conditions, were considered as inclusion criteria while more unstable forms of housing precarity such as homelessness or multiple moves in the last 12 months were considered as exclusion criteria, in order to maximize the likelihood of longitudinal follow-up.
> During the prenatal appointment, healthcare providers (HCPs; e.g., midwives, nurses, or obstetricians) used this questionnaire to determine eligibility for the ECAIL trial. (…)

The remaining files uploaded as part of the submission process (e.g., the STROBE checklist and ethics-related documents) were provided in accordance with to the journal's submission requirements and, to the best of our understanding, are intended for editorial review rather than publication as supplementary material.

1.6. 238-242: It is not standard to have thematic coding performed by a single researcher, especially for a team this size. The circumstances appear unique enough and the scope narrow enough that it doesn't endanger the paper, however if any other researchers or participants even briefly reviewed the content analysis (see 669-671), I strongly recommend elaborating it here.

We agree that the qualitative analytic process required further clarification. This study was conducted as part of a postdoctoral fellowship, with DP's qualitative work being supervised by PS throughout the study. While this supervisory process was not described in the original manuscript, we agree that it should be made explicit. As recommended, we have expanded the Methods section to describe the coding process in greater detail, including the iterative development of themes, the independent review of the coding and resulting themes by PS, and the consensus process used to resolve discrepancies (pages 11-12, lines 264-270):

> DP manually transcribed the focus group verbatim and imported it into NVivo software. A thematic content analysis (29) was then conducted. The transcript was coded by DP, and codes were iteratively grouped into broader themes reflecting implementation context, disruptions, adaptations, and perceived mechanisms influencing reach and engagement. The coding and resulting themes were independently reviewed by PS, and any discrepancies were discussed until consensus was reached. Illustrative quotations were then selected to exemplify the identified themes and subthemes and reflect dietitians' shared experiences of trial implementation during the pandemic.

1.7. 295-298 and Figure 4: I think this can be reported in text without giving an entire page to a diagram lacking significance.

We respectfully chose to retain Figure 4 in the main manuscript, as it presents one of the study's core quantitative findings. The stable distribution of the main reasons for nonparticipation before the pandemic and after study resumption is an important implementation finding, supporting the interpretation that the pandemic did not substantially alter the profile of nonparticipation among eligible women. Presenting these data graphically facilitates comparison between the two study periods. Should the manuscript be accepted, we anticipate that the figure size will be optimized during the journal's production process.

1.8. 319-330 This paragraph is an insightful reminder that even among "disadvantaged" patients, the minimum stability necessary to participate can and does exclude patients who are more disadvantaged. This section highlights an opening for future research on two topics: addressing the demands placed on patients who participate in research and addressing increases in social disadvantage in the years immediately following COVID lockdowns.

We appreciate the reviewer's insightful interpretation and agree that these findings highlight important avenues for future research among socially disadvantaged populations. These broader implications are discussed in the Perspectives, Limitations and Strengths, and Conclusion sections. We have also strengthened the Discussion in response to Comment 1.19 by highlighting that the minimum level of stability required to participate in longitudinal intervention studies may inadvertently exclude some women experiencing social vulnerability. In addition, we now explicitly emphasize the need for future research to explore strategies to reduce participation burden and develop implementation approaches that better reach families experiencing

the highest levels of social disadvantage (page 22, lines 453-457):

> These findings also suggest that the minimum level of stability required to participate in longitudinal intervention studies may inadvertently exclude some women experiencing social vulnerability, an issue that warrants further investigation. Future research should explore strategies to reduce participation burden and develop implementation approaches that better reach families experiencing the highest levels of social disadvantage.

1.9. 337 no *significant difference

We have added the word "significant", as suggested by the Reviewer (page 18, line 374).

1.10. 346-348: at no point are these "remote solutions" introduced or discussed, let alone dieticians' reasons for rejecting them.

We agree that this point was insufficiently described. We have clarified what was meant by "remote solutions" and added a brief explanation of why dietitians considered them incompatible with the key features of the ECAIL intervention (page 18, lines 383–389).

> Despite these obstacles, dietitians remained committed to maintaining the study's home-visit model, considering that remote alternatives, such as telephone or video counseling, were not compatible with the face-to-face nature of the study, which relied on interactions with both parents and children and the delivery of tailored nutritional counselling. Their input was instrumental in designing the subsequent adaptations, as they took part in discussions surrounding implementation under new constraints. Nevertheless, they maintained contact with participating families through telephone calls and newsletters while preparing for the resumption of home visits.

1.11. 353: where was ECAIL prioritized before resumption? what else was it competing with for clinical attention? Table 5 includes a quotation implying that a lot of other programs were still on pause when ECAIL resumed - this link should be explored in discussion and possibly earlier.

We agree that the wording could be clarified. Our intention was to indicate that, following the first lockdown, ECAIL was authorized to resume before many other clinical studies at Lille University Hospital because of the social vulnerability of its participants. We have revised the text accordingly:

> Results (page 18, lines 392-393) : The ECAIL study was authorized to resume before many other clinical studies at Lille University Hospital because of the social vulnerability of its participants.

> Discussion (pages 22-23, lines 466-470): This shift may have resulted from the prioritization of the ECAIL trial after study resumption, which likely created favorable conditions for implementation. As highlighted in the qualitative findings, ECAIL was authorized to resume before many other clinical studies because of the social vulnerability of its participants, illustrating an equity-oriented approach.

1.12. 354-359: say more about the impact on social isolation (perhaps in discussion) and mask-wearing (protocols, tensions, and timing of relaxation of masking standards), and ideally confer these conditions against contemporary research (about social isolation and masking, respectively).

We agree that these findings deserved further contextualization. We have therefore expanded the Discussion to emphasize that the resumption of home visits may have helped mitigate the social isolation experienced by socially disadvantaged families during successive lockdowns, as follows:

> Page 24, lines 502–504: This may partly explain why mothers perceived the resumption of home visits as particularly valuable after successive lockdowns, when opportunities for social interaction had been substantially reduced.

> Page 25, lines 543-544: They also suggest that home visits and one-to-one interactions may strengthen participation and commitment, despite constraints on face-to-face contact.

Regarding mask-wearing, our intention was not to discuss the broader public health implications of masking policies, but rather to interpret its role as an implementation adaptation within the ECAIL trial. We therefore chose to keep the Discussion focused on implementation processes and outcomes directly supported by our

data.

1.13. 359-360: what was the risk assessment questionnaire, how was it implemented, and did its implementation change before the end of the trial?

The pre-visit COVID-19 risk assessment questionnaire is described in the Methods section (pages 7-8, lines 161-169). Briefly, before each home visit, dietitians contacted participants by telephone to assess recent COVID-19 symptoms and contact with confirmed cases. Depending on the responses, visits could be maintained or postponed after consultation with a physician when necessary. This procedure was introduced upon trial resumption after the first lockdown and remained in place until 10 August 2022 (Figure 1).

1.14. Discussion: why was in-person delivery of information more common after lockdown than before? any theories?

Our interpretation is that the higher frequency of face-to-face delivery of study information after study resumption reflected organizational changes introduced at that time. Specifically, the prioritization of the ECAIL trial created favorable conditions for implementation, reflecting an equity-oriented approach, while reinforcing coordination between dietitians and healthcare providers and increasing opportunities for direct contact with eligible women during prenatal appointments. We have clarified this interpretation in the Discussion (pages 22-23, lines 464-476):

> From an *Adoption* perspective, this was likely associated with a higher frequency of face-to-face delivery of study information at the maternity ward, rather than telephone contact. This shift may have resulted from the prioritization of the ECAIL trial after study resumption, which likely created favorable conditions for implementation. As highlighted in the qualitative findings, ECAIL was authorized to resume before many other clinical studies because of the social vulnerability of its participants, illustrating an equity-oriented approach. Direct interactions between dietitians and women (and their partners, when present) may have facilitated participant recruitment more effectively than delayed, impersonal phone calls, although this depended on HCPs contacting the dietitians during prenatal appointments. In this context, regular meetings between the ECAIL team and HCPs likely supported sustained and synergistic collaboration despite pandemic-related pressures on hospital services. Together, quantitative and qualitative findings support the interpretation that coordination between dietitians and HCPs was strengthened during the early post-resumption period (P1).

1.15. 366-371: This overstates the authors' case; the present study may contribute original research on these topics but it is far from proven.

We acknowledge that the opening of the Discussion could be interpreted as overstating the implications of our findings. We have therefore revised this paragraph throughout to adopt a more cautious wording, emphasizing that our findings suggest how trial implementation evolved across the COVID-19 pandemic and the subsequent cost-of-living crisis, rather than supporting broader conclusions beyond the scope of the study. We also tempered our interpretation of implementation robustness to better reflect the observational nature of the study and the temporal variation observed across the post-resumption period (page 20, lines 406-411):

> This study of an interdisciplinary public health intervention among socially disadvantaged families provides implementation-relevant insights into how trial implementation evolved across the COVID-19 pandemic and the subsequent cost-of-living crisis. Interpreted through a RE-AIM lens (19, 20), our findings suggest that these contextual shocks were associated with an increase in the proportion of women meeting the trial eligibility criteria, without compromising provider *Adoption* or *Implementation*, and may even have reinforced engagement during key phases of delivery.

1.16. 380-400: Discussion is too late to introduce this profile of the community involved; it should go much earlier, under "study design and participants".

We agree that readers should be introduced earlier to the socioeconomic context of the study setting. We have therefore added a brief description of the Hauts-de-France region in the Introduction (page 4, lines 89-90).

> We seek to address this gap by analyzing multiple sources of evidence generated within the prEgnanCy and eArly Childhood nutrItion triaL (ECAIL), launched in 2017 in northern France (23), a region characterized by high levels of socioeconomic disadvantage (24).

In addition, in response to Comment 1.3, we substantially shortened and refocused the corresponding subsection of the Discussion, retaining only the contextual elements most directly relevant to interpreting the consistently high eligibility rate observed in the trial.

1.17. 405-407: not relevant

This sentence has been removed as part of the revisions made in response to Comment 1.3, which substantially streamlined this section of the Discussion.

1.18. 411-413: authors have not reported when and how health restrictions were lifted, nor defined "exceptional support measures", see also 476-477.

We agree that the expression "exceptional support measures" was too vague for an international readership. We have therefore replaced it with "exceptional government financial support introduced during the COVID-19 pandemic" to make the meaning clearer for an international audience (page 22, lines 445-446).

Regarding the timing of the lifting of health restrictions and the final lockdown (lines 476–477 of the original manuscript), these events are already presented in the study timeline (Figure 1).

1.19. 419-423: say more

Thank you for this suggestion. We interpreted this comment together with your broader remark (Comment 1.8) regarding the exclusion of some socially vulnerable women despite the trial targeting disadvantaged families. We therefore strengthened the implications of this finding by adding the following sentence to the end of the paragraph (page 22, lines 450-459):

> This worsening socioeconomic context is also reflected in our analysis of women ineligible for the ECAIL trial despite experiencing social vulnerability. These women faced increasing housing precarity and instability, as well as heightened social isolation, consistent with previous findings from the Haut-de-France region (42). These findings also suggest that the minimum level of stability required to participate in longitudinal intervention studies may inadvertently exclude some women experiencing social vulnerability, an issue that warrants further investigation. Future research should explore strategies to reduce participation burden and develop implementation approaches that better reach families experiencing the highest levels of social disadvantage.
> Taken together, these results underscore how multidimensional crises such as the COVID-19 pandemic can intensify pre-existing vulnerabilities, highlighting the need for resilient intervention designs.

1.20. 427-4460: this section seems heavy on analysis, which should come before discussion but after and separate from Methods. It also elaborates on relevant topics that were not established under Methods (such as concerns over masking).

Our intention in this section was to interpret the quantitative and qualitative findings jointly, as appropriate in the Discussion, rather than to introduce new results. Nevertheless, we have revised the wording throughout the paragraph to more clearly distinguish observations from interpretations by adopting more cautious language (e.g., "are consistent with", "may", "support the interpretation that") and by making explicit that the discussion of protective measures and coordination between dietitians and healthcare providers is grounded in the qualitative findings (page 22-23, lines 462-476):

> Participation in the ECAIL trial increased slightly during the post-resumption P1 and P2 subperiods. From an *Implementation* perspective, these findings are consistent with the dieticians' perception that protective measures, including mask wearing, did not substantially interfere with screening interviews. From an *Adoption* perspective, this was likely associated with a higher frequency of face-to-face delivery of study information at the maternity ward,

rather than telephone contact. This shift may have resulted from the prioritization of the ECAIL trial after study resumption, which likely created favorable conditions for implementation. As highlighted in the qualitative findings, ECAIL was authorized to resume before many other clinical studies because of the social vulnerability of its participants, illustrating an equity-oriented approach. Direct interactions between dietitians and women (and their partners, when present) may have facilitated participant recruitment more effectively than delayed, impersonal phone calls, although this depended on HCPs contacting the dietitians during prenatal appointments. In this context, regular meetings between the ECAIL team and HCPs likely supported sustained and synergistic collaboration despite pandemic-related pressures on hospital services. Together, quantitative and qualitative findings support the interpretation that coordination between dietitians and HCPs was strengthened during the early post-resumption period (P1).

1.21. 434-435: the favorable conditions are demonstrated, but not the equity-oriented approach. Authors must introduce this approach (background, importance, relevance to stakeholders) much earlier.

We agree that this point required further clarification. In the revised manuscript, we now explain in the qualitative Results section that the ECAIL trial was authorized to resume before many other clinical studies at Lille University Hospital because of the social vulnerability of its participants (page 18, lines 392-393).

> The ECAIL study was authorized to resume before many other clinical studies at Lille University Hospital because of the social vulnerability of its participants.

We have also revised the corresponding paragraph in the Discussion to make explicit that this prioritization likely created favorable conditions for implementation and may illustrate an equity-oriented approach, as follows (pages 22-23, lines 466-474).

> This shift may have resulted from the prioritization of the ECAIL trial after study resumption, which likely created favorable conditions for implementation. As highlighted in the qualitative findings, ECAIL was authorized to resume before many other clinical studies because of the social vulnerability of its participants, illustrating an equity-oriented approach. Direct interactions between dietitians and women (and their partners, when present) may have facilitated participant recruitment more effectively than delayed, impersonal phone calls, although this depended on HCPs contacting the dietitians during prenatal appointments. In this context, regular meetings between the ECAIL team and HCPs likely supported sustained and synergistic collaboration despite pandemic-related pressures on hospital services.

1.22. 437-446: repetitive of earlier sections, though this is the more appropriate place for it.

Following the revisions made throughout the Discussion, several overlapping points have been streamlined or removed from earlier sections. This paragraph now serves as the main location where we interpret the temporal evolution of participation and its possible explanations, thereby reducing repetition while keeping the discussion focused on the implementation findings.

1.23. 448: In my field, we usually call this "recommendations"

We believe the reviewer is referring to the subsection entitled "Follow-up". In this context, "Follow-up" refers to participant follow-up within the ECAIL trial rather than to recommendations or future directions. We therefore believe that this heading appropriately reflects the content of the subsection and have retained it.

1.24. 455-463: this passage conveys the most significant contribution to research, and it feels buried under everything else. What can be trimmed to give this point more room for discussion?

We appreciate this comment and agree that these methodological implications represent one of the main contributions of our study. Rather than expanding this section further, we substantially streamlined earlier sections of the Discussion, thereby giving greater prominence to this methodological contribution. We also added a sentence emphasizing the particular value of home visits following successive lockdowns, as follows (pages 23-24, lines 492-504):

> These observations have significant methodological implications and highlight the value of ongoing relational work between follow-up visits. This requires additional human resources and research time, particularly when working with socially disadvantaged populations, who are known to be "hard-to-survey" (21, 22). However, this investment may improve the efficiency of trial implementation by reducing unnecessary trips and facilitating visit rescheduling, thus saving time and subsequent manhour costs in the long term, while also enhancing adherence to the study protocol, participant commitment, and data quality by limiting missed visits. More broadly, our findings reinforce the relevance of home-based delivery for reaching and engaging populations facing social adversity, who often have lower utilization of health care and community services and experience greater social isolation (43, 44), while facilitating one-on-one interactions that foster trust between providers and mothers and enable more personalized and context-sensitive interventions (18). This may partly explain why mothers perceived the resumption of home visits as particularly valuable after successive lockdowns, when opportunities for social interaction had been substantially reduced.

1.25. 465-473: messy and unhelpful

We believe this section is important because the present manuscript contributes to the process evaluation of this complex public health intervention. The implementation findings presented here provide important context for interpreting the forthcoming effectiveness analyses and the trial's external validity, consistent with established process evaluation frameworks (46–49). We nevertheless revised the wording to improve the overall readability of the section (page 24, lines 510-511).

> These findings provide important insights for interpreting the trial's external validity and the potential generalizability of its future effectiveness findings.

1.26. 476: was it a group interview or a focus group? Be consistent with terms. Also, this delay should be mentioned above, under qualitative analysis.

Although the term "group interview" is sometimes used as a generic term encompassing focus groups, we agree that using a single term throughout the manuscript improves clarity. We have therefore harmonized the terminology and now use "focus group" consistently. Regarding its timing, this information was already provided in the Methods section: "A researcher in psychosocial sciences (DP) conducted an in-person focus group in May 2023 at the Lille University Hospital..." (page 10, line 223). Its timing relative to the successive COVID-19 lockdowns and study resumption can also be inferred from the study timeline presented in Figure 1. We therefore considered that no further changes to the manuscript were necessary.

1.27. 669 and elsewhere: authors keep saying "interdisciplinary" when I think they mean "mixed method", i.e. qualitative and quantitative analysis. "Interdisciplinary" may also apply (if not only your team comprises researchers from multiple fields, but also your approaches to patients and data), but that is not evident in this draft.

We agree that the present manuscript reports findings from a convergent mixed-methods implementation study, and we have revised the title and the Authors' contribution section to make this design more explicit. The term "interdisciplinary" is retained where it refers to the broader ECAIL project and intervention, which brought together researchers from multiple disciplines, clinicians, dietitians, and community stakeholders to co-create and implement the intervention. It is therefore not intended to describe the mixed-methods design itself. We have also revised the wording in the Discussion to avoid any ambiguity between the interdisciplinary nature of the ECAIL project and the convergent mixed-methods design of the present study (page 20, lines 406-408):

> This study of an interdisciplinary public health intervention among socially disadvantaged families provides implementation-relevant insights into how trial implementation evolved across the COVID-19 pandemic and the subsequent cost-of-living crisis.

Reviewer #2:

I appreciate the opportunity to review this study, which addresses a highly relevant and timely topic. Numerous research projects experienced interruptions or modifications due to the COVID-19 pandemic; however, this work stands out for having successfully maintained the implementation of the intervention and documented its effects across different time periods. Among its main strengths are the availability of data collected before, during, and after the pandemic, as well as the integration of quantitative and qualitative methods, providing a broader understanding of implementation processes.

We thank the reviewer for this positive and encouraging assessment of our work and for recognizing both the originality of the study and the strengths of our mixed-methods approach.

With the aim of contributing to the strengthening of the manuscript, I offer the following comments:

Methodology

2.1. The quantitative findings could be strengthened through multivariable analyses. The reported associations are based primarily on chi-square tests, which limits the ability to evaluate the independent contribution of the different variables examined.

The primary objective of this study was to describe implementation-related indicators across study periods, in line with the RE-AIM framework, rather than to identify independent predictors of these outcomes. Accordingly, the quantitative analyses were intentionally descriptive, with formal statistical comparisons performed where appropriate. We have clarified this objective in the Methods section (page 11, lines 247-250). While multivariable analyses could be informative for addressing different research questions, they were beyond the scope of the present implementation study.

> The quantitative analysis primarily consisted of describing implementation-related indicators across study periods, in line with the RE-AIM framework. Where appropriate, formal statistical comparisons were performed to assess differences either between the two main study periods (pre-pandemic vs post-study resumption) or across the four study periods (P0–P3), depending on the analysis presented.

2.2. There is a possible discrepancy in the operationalization of the Adoption dimension of the RE-AIM framework. Traditionally, this dimension refers to the extent to which organizations, institutions, or professionals adopt an intervention. In the present study, adoption appears to be assessed mainly through the mode of delivery of study information to participants. It would therefore be helpful to provide a stronger justification for this methodological decision or reconsider its classification within the conceptual framework.

We agree that, within the RE-AIM framework, *adoption* generally refers to the uptake of an intervention by organizations or professionals. In the present study, the mode of delivery of study information was not considered an *adoption* outcome per se. Rather, the frequency of face-to-face delivery was used as an indirect indicator of HCP engagement and, therefore, *adoption*, because it required healthcare providers to actively contact an ECAIL dietitian during the participant screening process. We have clarified this rationale in the Methods section (page 9, lines 195-197).

> The frequency of face-to-face delivery of study information was also interpreted as an indirect indicator of HCP engagement and, therefore, *Adoption*, because it required HCPs to actively contact an ECAIL dietitian during the participant screening procedure.

2.3. There appears to be a potential conceptual overlap between the inclusion criterion of "precarious housing" and the exclusion criteria related to homelessness, emergency accommodation, hotel housing, or housing instability. As currently presented, some participants could potentially meet both inclusion and

exclusion criteria simultaneously. The authors should clarify the operational definitions used during the screening process and explain how potentially ambiguous situations were classified.

Although the operational definitions were already provided in Table 1 and in the eligibility questionnaire (Supplementary Document 1), we agree that the underlying rationale could be made clearer. The distinction was between women experiencing precarious but sufficiently stable housing conditions, who were eligible, and women experiencing homelessness or marked residential instability, for whom the longitudinal follow-up required by the trial was considered unfeasible. We have clarified this point in the Methods section (page 5, lines 124-127).

> Some forms of precarious housing, such as substandard living conditions, were considered as inclusion criteria while more unstable forms of housing precarity such as homelessness or multiple moves in the last 12 months were considered as exclusion criteria, in order to maximize the likelihood of longitudinal follow-up.

Results

2.4. Regarding the statement that "contextual shocks expanded the pool of eligible individuals," although the observed difference in eligibility reached statistical significance, the absolute increase was approximately four percentage points. It would be advisable to complement the presentation of results with absolute differences to facilitate assessment of their practical significance.

In response to Reviewer 1's comments, we substantially revised the opening paragraph of the Discussion to adopt a more cautious interpretation of this finding. The revised wording now emphasizes that the contextual shocks were associated with an increase in the proportion of women meeting the trial eligibility criteria, rather than using a stronger formulation (page 20, lines 406-411). We believe this revised wording more appropriately reflects both the magnitude and interpretation of the observed difference. Although the absolute increase was 3.9 percentage points, this corresponds to approximately 100 additional women meeting the eligibility criteria during the post-resumption period compared with what would have been expected had pre-pandemic eligibility rates remained unchanged. Given the implementation focus of our study, we consider this difference to be of practical importance.

> This study of an interdisciplinary public health intervention among socially disadvantaged families provides implementation-relevant insights into how trial implementation evolved across the COVID-19 pandemic and the subsequent cost-of-living crisis. Interpreted through a RE-AIM lens (19, 20), our findings suggest that these contextual shocks were associated with an increase in the proportion of women meeting the trial eligibility criteria, without compromising provider *Adoption* or *Implementation*, and may even have reinforced engagement during key phases of delivery.

2.5. It would also be useful to include basic sociodemographic characteristics of the study population, such as age, marital status, educational level, or other relevant variables, as these would contribute to a better characterization of the participants and provide additional context for interpreting the findings.

We agree that a detailed description of participants' sociodemographic characteristics is important for interpreting the effectiveness of the intervention and will be reported in the main trial publication, in accordance with CONSORT recommendations. However, the primary objective of the present manuscript was to describe implementation outcomes and their evolution over time within the RE-AIM framework, rather than to report trial effectiveness or participant baseline characteristics. To avoid unnecessary duplication across publications, we therefore chose not to include a full baseline description in this implementation-focused manuscript. Nevertheless, the key dimensions of social vulnerability used to determine eligibility are described in Table 1, and their distributions are presented in the Results (e.g., Table 2), as these characteristics were directly relevant to the implementation questions addressed in this study.

Discussion

2.6. I suggest explicitly acknowledging, among the study limitations, the potential bias arising from the fact that the qualitative component was based exclusively on a focus group of dietitians. The inclusion of perspectives from other stakeholders, such as participants or clinical staff, could have enriched the understanding of implementation processes.

We agree that relying exclusively on a focus group with dietitians represents a limitation of the present study and have now acknowledged this explicitly in the Limitations section (page 24, lines 517-519).

> One limitation of this study is that the qualitative component relied exclusively on a focus group with dietitians, whose perspectives may not fully reflect those of other stakeholders involved in trial implementation.

At the same time, we have further clarified why this perspective also constitutes a strength, as dietitians were the frontline implementers of the trial and were directly involved in participant recruitment, follow-up, and data collection. Their perspectives therefore provided unique insights into the implementation processes during the successive crises (page 25, lines 534-538).

> Importantly, the inclusion of dietitians' perspectives represents a further strength because they were the frontline implementers of the trial and directly involved in participant recruitment, follow-up and data collection. Their perspectives, which are still relatively rarely captured in implementation research, provided valuable insights into day-to-day implementation during the successive crises.

Conclusions

2.7. It is recommended that the authors moderate statements that imply causal relationships between the pandemic and the observed changes in eligibility or participation. Given that this is an observational and descriptive study, conclusions should be framed primarily in terms of temporal associations rather than causal effects.

We agree that causal interpretations should be avoided in the context of this observational and descriptive implementation study. In response to this comment, we have revised the Conclusion to consistently use more cautious language, framing the observed changes as temporal associations rather than causal effects (e.g., "was associated with", "may influence", "may have helped", etc.; page 26, lines 547-559). We believe these revisions more accurately reflect the observational and descriptive nature of the study while preserving its main implementation messages.

> The social impact of both the pandemic and the cost-of-living crisis, often described as the "second health emergency" (5), was associated with a higher eligibility rate among socially disadvantaged families in the ECAIL trial, while participation remained stable. These findings provide important insights into how successive crises may influence participation in programs designed to mitigate social disadvantage. In the ECAIL trial, the collaborative foundations of this co-created intervention may have helped sustain both stakeholder and participant engagement despite increased workload and organizational strain experienced within hospitals during the successive crises. Moreover, home visits and telephone calls between follow-ups were perceived as effective implementation strategies, not only for reaching families and supporting adherence, but also for mitigating social isolation among participants. Overall, these findings suggest that interventions co-designed with stakeholders and target populations, and supported by appropriate implementation strategies, may remain broadly resilient during major external disruptions, highlighting the value of flexible, context-sensitive implementation approaches in a context of rising poverty and ongoing geopolitical and economic instability.

I hope these comments are helpful to the authors in revising and strengthening the manuscript.

We sincerely thank the reviewer for these thoughtful and constructive comments. We believe they have helped us improve the clarity and methodological rigor of the manuscript.

Reviewer #3:

3.1. It is not entirely clear to me what is the question this paper is trying to answer and why is it so long. The same information (more people were eligible to participate in this intervention after rather than before the pandemic) could have been more succinctly presented in a letter to the editor type format. It would be better if this paper asked some questions that the authors were answering. As it stands it's more like a progress report of an ongoing study.

We appreciate the reviewer's overall assessment. We agree that, in the original version, the research question and the methodological framing of the study were not sufficiently explicit. In response to this comment, and to related comments from the other reviewers, we substantially revised the Title, Abstract, Introduction, Methods, and opening of the Discussion to better define the research question addressed by the study, clarify the conceptual framework (RE-AIM) and the convergent mixed-methods design, and more explicitly position the manuscript as an implementation study. In addition, we substantially streamlined the Discussion by removing redundant material and sharpening the interpretation of the findings. We believe these revisions have considerably improved the focus, coherence, and readability of the manuscript, and we hope they make its objectives, contribution, and added value clearer to readers.

Reviewer #4:

Abstract

4.1. Line 32–34 and title. The framing that the pandemic and cost-of-living crisis "shaped" reach and engagement implies causation. The design is a single-site before/after comparison without a control group, so it can describe associations between time periods and outcomes but cannot attribute change specifically to these crises. Consider associational language here and throughout (e.g., "changes in reach and engagement observed across the pandemic and cost-of-living period").

We agree that the original wording could be interpreted as implying a causal relationship. In response to this comment, we revised both the title and the Abstract to adopt more cautious, associational language, referring to changes observed across the COVID-19 pandemic and subsequent cost-of-living crisis rather than changes attributed to these events. These revisions are consistent with similar changes made throughout the manuscript in response to related comments from the other reviewers.

> Title: Reach and engagement in the ECAIL trial targeting socially disadvantaged families across the COVID-19 pandemic and cost-of-living crisis: a mixed-methods implementation study.
>
> Abstract (page 2, lines 32-34): This study compared eligibility and participation before the pandemic and after resumption, and examined how implementation and reach evolved across the pandemic and subsequent cost-of-living crisis.

4.2. Line 44. Participation is reported as n=443 here and in the main text (lines 115, 257, 284), but Table 2 reports follow-up n=444. Please reconcile and state the enrolled N consistently.

We thank the reviewer for identifying this inconsistency. The discrepancy resulted from typographical errors in Table 2, which have now been corrected. The number of enrolled participants is now reported consistently throughout the manuscript and in Table 2.

4.3. Line 46–47. "the introduction of a pre-visit COVID-19 questionnaire reduced missed appointments." Because the pre-visit telephone call works by confirming presence before a trip is made, a reduction in wasted trips is partly definitional rather than an independent effect; please phrase more cautiously (see Results comment 7).

We agree that this wording was too strong for the Abstract. We have revised the sentence to use more cautious associational language and to align it more closely with the implementation indicator measured (page 2, lines 47-48).

> After resumption, the introduction of a pre-visit COVID-19 questionnaire was associated with fewer failed home visits.

4.4. The abstract notes that eligibility increased significantly but does not note that the distribution of vulnerability indicators among eligible women did not change. This distinction matters for interpretation (more eligible women, but not a more vulnerable eligible group); consider making it explicit.

We agree that this distinction is important for the interpretation of our findings. We believe, however, that the current wording of the Abstract already conveys this point by explicitly stating that, although eligibility increased after study resumption, "the distribution of vulnerability criteria remained similar across periods," indicating that the profile of eligible women did not substantially change. We therefore retained the current wording.

Introduction

4.5. Lines 65–79. Well-referenced but long on general macroeconomic context. Consider tightening to foreground the specific gap (implementation outcomes under crisis for hard-to-reach groups), which is the genuine contribution.

We agree that the introduction should more rapidly foreground the specific research gap addressed by the study. We therefore streamlined the description of the broader socioeconomic context while retaining the key contextual elements necessary to understand the successive crises experienced by socially disadvantaged populations (page 3, lines 68-76).

> In 2020, the COVID-19 pandemic triggered a massive health crisis worldwide, accompanied by prolonged economic and social repercussions. Following the initial lifting of pandemic-related health restrictions, subsequent geopolitical shocks, including the invasion of Ukraine, fueled inflation and rising living costs across Europe (1). Although governments implemented exceptional support measures, low-income households experienced a disproportionate loss of purchasing power, particularly for food, transportation, and housing, further exacerbating pre-existing social, economic, and health inequalities (2-10). These successive health and cost-of-living crises have therefore disproportionately affected populations already experiencing social disadvantage, illustrating a cumulative disadvantage often described as the "poverty penalty" (6).

This allowed us to place greater emphasis on the knowledge gap in implementation science addressed by the present study, namely the limited understanding of how implementation outcomes such as reach, adoption, and implementation are sustained, adapted, or disrupted under crisis conditions, particularly for interventions targeting socially disadvantaged populations.

4.6. Lines 86–90. Please state up front that only Reach, Adoption, and Implementation are assessed (not Effectiveness or Maintenance), so readers calibrate expectations early.

We agree that the scope of the present study should be stated more explicitly early in the Introduction. We have therefore added a sentence clarifying that, consistent with the RE-AIM framework, this implementation study focuses specifically on the Reach, Adoption, and Implementation dimensions, whereas the Effectiveness and Maintenance dimensions are beyond its scope and will be reported separately (page 4, lines 96-98).

> Consistent with the RE-AIM framework, the present implementation study focuses specifically on the Reach, Adoption, and Implementation dimensions, whereas Effectiveness and Maintenance dimensions are beyond its scope and will be reported separately.

4.7. Lines 91–100. The aims are clear, but Aim (ii) again uses causal phrasing ("shaped implementation"); consider aligning with the descriptive design.

We agree that the original wording could be interpreted as implying a causal relationship. We have therefore revised the second study aim to adopt a more descriptive formulation. Rather than referring to how the COVID-19 pandemic and the subsequent cost-of-living crisis shaped implementation, the revised objective now states that the study explores implementation processes, including delivery adaptations and stakeholder engagement, across the pandemic and subsequent cost-of-living crisis (page 4, lines 100-102).

> (…) and (ii) explore implementation processes, including delivery adaptations and stakeholder engagement, across the pandemic and subsequent cost-of-living crisis.

Methods

4.8. Lines 180–184. The eligibility rate denominator is the number of questionnaires completed and returned to dietitians. This denominator is itself shaped by the prescreening phase (lines 135–137) and by reception/HCP behavior, both of which may have changed over six years. If prescreening became more targeted after resumption, the eligibility rate would rise independently of any real change in the underlying population. Please report the number of questionnaires distributed and returned per period, and discuss how changes in prescreening/distribution could affect the eligibility comparison.

We agree that changes in prescreening practices could theoretically influence the observed eligibility rate. However, the prescreening procedure, implemented by the administrative staff, remained unchanged throughout the study and was based on a limited number of objective administrative criteria (e.g., age and residential address; see footnote of Table 1), leaving little room for subjective selection by healthcare providers. The detailed eligibility assessment was subsequently performed using the same eligibility questionnaire, which was self-completed by pregnant women in the waiting room (page 5, lines 123-124). Moreover, if prescreening had become substantially more targeted after study resumption, one might expect changes in the distribution of social vulnerability criteria among eligible women. In contrast, the profile of eligible women remained remarkably stable across study periods, suggesting that the observed increase in eligibility was unlikely to be explained solely by changes in prescreening practices (pages 12-13, lines 290-294). We have therefore not added additional analyses on questionnaire distribution.

4.9. Lines 175–178. The RE-AIM operationalization needs sharpening. "Delivery modalities" are mapped to both Adoption and Implementation, which is confusing, and mapping in-person versus telephone delivery onto Adoption is non-standard (Adoption usually refers to setting/staff uptake). Please justify the mapping and avoid assigning one indicator to two dimensions. Relatedly, the representativeness comparisons (participants vs nonparticipants vs ineligible on vulnerability) are arguably the strongest Reach contribution and could be framed explicitly as such.

We agree that the operationalization of the RE-AIM dimensions should be made clearer. We have therefore revised this section to distinguish more explicitly between the implementation indicator itself (delivery modalities) and its interpretation within the RE-AIM framework (page 9, lines 191-197).
Delivery modalities are now presented as indicators of *Implementation*, whereas the frequency of face-to-face delivery of study information is described as an indirect indicator of HCP engagement and, therefore, *Adoption*, because it required healthcare providers to actively contact an ECAIL dietitian during the participant screening procedure. We also agree that the comparison of social vulnerability profiles across eligible, ineligible, participating, and nonparticipating women represents an important contribution to the *Reach* dimension. To make this clearer, we have revised the description of the Reach indicators in the Methods section to explicitly include these comparisons.

> **RE-AIM indicators and their measurement**
> Drawing on the RE-AIM framework, the following indicators were selected: eligibility, participation, and the social vulnerability profiles of eligible, ineligible, participating, and nonparticipating women as indicators of *Reach*; delivery

> modalities and follow-up completion and adaptations as indicators of *Implementation* (19, 20). The frequency of face-to-face delivery of study information was also interpreted as an indirect indicator of HCP engagement and, therefore, *Adoption*, because it required HCPs to actively contact an ECAIL dietitian during the participant screening procedure (19, 20).

4.10. Lines 168–172 and Fig 1. Please state explicitly that the suspension window (March–September 2020) is excluded from both periods. More importantly, Fig 1 shows that P1 (Sept 2020–Aug 2021) contains two further lockdown periods (Oct–Dec 2020 and Apr–Jun 2021), so P1 is not a clean "post-resumption" period; this complicates interpreting P1 as early recovery and should be addressed.

We agree that this point should be made more explicit. The suspension period (March to September 2020) was excluded from all analyses. We have also clarified in the Methods section that the post-study resumption period encompassed successive phases of the COVID-19 pandemic and the progressive relaxation of public health measures, rather than a period free of COVID-19 restrictions (page 8, lines 180-189). This distinction is important because the objective of the study was precisely to examine implementation throughout these successive crisis phases following study resumption, rather than during a simple post-pandemic recovery period.

> We defined two periods, namely pre-pandemic (March 2017 to March 2020; P0) and post-study resumption (September 2020 through April 2023). As shown in **Fig 1**, the post-resumption period encompassed successive phases of the COVID-19 pandemic and the progressive relaxation of public health measures. These phases corresponded to successive changes in national public health restrictions and hospital infection-control procedures that characterized the implementation context of the ECAIL trial, including mandatory masking and the study-specific COVID-19 safety protocol introduced upon trial resumption. To capture temporal variation in implementation conditions, the post-resumption period was segmented into three approximately one-year periods (September-August). This segmentation also ensured comparable seasonal coverage across periods. The final period was shorter due to the end of screening in April 2023 (**Fig 1**; P1, P2, and P3).

4.11. Lines 182–184. Using in-person delivery as a proxy for HCP engagement is reasonable but indirect; the decline from P1 to P3 could also reflect dietitian availability, staffing, or scheduling. Please acknowledge these alternatives where the indicator is interpreted.

We agree that the mode of study information delivery represents an indirect rather than a direct measure of HCP engagement. In response to this comment, we have clarified in the Methods section that face-to-face delivery of study information was interpreted as an indirect indicator of HCP engagement because it required healthcare providers to actively contact an ECAIL dietitian during the participant screening procedure:

> Pages 5-6, lines 132-134): Importantly, the provision of study information face to face by dietitians, rather than exclusively by telephone, served as an indirect indicator of HCP engagement with the screening procedure.

> Page (page 9, lines 195-197): The frequency of face-to-face delivery of study information was also interpreted as an indirect indicator of HCP engagement and, therefore, *Adoption*, because it required HCPs to actively contact an ECAIL dietitian during the participant screening procedure (19, 20).

This interpretation is further supported by both the qualitative findings and the strong association observed between face-to-face delivery of study information and participation (Table 2).

4.12. Lines 204–242. Several qualitative reporting elements are missing. Transcription, coding, and thematic analysis appear to have been done by a single researcher (DP); please state whether any second coding, peer debriefing, or reflexivity procedures were used. Because participants' reactions are reported secondhand by dietitians, please make clear (here and in Results/Discussion) that no participant data were collected directly in this component. A COREQ (or SRQR) checklist should accompany STROBE given the focus-group component.

We agree that additional methodological details were needed to strengthen the reporting of the qualitative component. In response to comments from the reviewers, we have expanded the description of the qualitative analysis to clarify the coding process, the independent review of the coding and resulting themes by a second researcher (PS), and the consensus procedure used to resolve discrepancies (pages 11-12, lines 264-270).

> DP manually transcribed the focus group verbatim and imported it into NVivo software. A thematic content analysis (29) was then conducted. The transcript was coded by DP, and codes were iteratively grouped into broader themes reflecting implementation context, disruptions, adaptations, and perceived mechanisms influencing reach and engagement. The coding and resulting themes were independently reviewed by PS, and any discrepancies were discussed until consensus was reached. Illustrative quotations were then selected to exemplify the identified themes and subthemes and reflect dietitians' shared experiences of trial implementation during the pandemic.

We have also clarified that this qualitative component was designed to capture the perspectives of dietitians as frontline implementers of the trial. Consequently, participants' experiences reported in the Results and Discussion reflect dietitians' accounts rather than data collected directly from participating women:

> Methods (page 10, lines 231-236): Second, dietitians were identified *a priori* as the most relevant stakeholders for this qualitative component, given their central role in trial implementation and their sustained engagement with participants throughout the study. Third, as the dietitians shared a common implementation context, a focus group facilitated the collection of shared perspectives, experiences, and adaptations. This approach was particularly suited to capturing local contextual influences on intervention delivery.

> Discussion (page 24, lines 517-519): One limitation of this study is that the qualitative component relied exclusively on a focus group with dietitians, whose perspectives may not fully reflect those of other stakeholders involved in trial implementation.

> Discussion (page 25, lines 534-538): Importantly, the inclusion of dietitians' perspectives represents a further strength because they were the frontline implementers of the trial and directly involved in participant recruitment, follow-up and data collection. Their perspectives, which are still relatively rarely captured in implementation research, provided valuable insights into day-to-day implementation during the successive crises.

Regarding the reporting guidelines, the present study is a convergent mixed-methods implementation study rather than a standalone qualitative study. We therefore followed the STROBE guidelines for the observational component while expanding the reporting of the qualitative methods in response to the reviewers' comments. We did not adopt the COREQ or SRQR checklists because the qualitative component was embedded within a broader mixed-methods implementation study rather than constituting a standalone qualitative investigation.

4.13. Lines 233 and 245–246. The manuscript appears to report two-period χ2 tests in the tables/text and four-period χ2 tests in the figures (e.g., the same indicators carry different P-values in Table 2 vs Fig 3). Please state this explicitly in the Analysis section, label each table and figure with the comparison performed, and indicate whether any adjustment for multiple comparisons was made given the number of tests across Tables 2–4 and Figs 2–6.

We agree that the statistical comparisons underlying the tables and figures should be made clearer. We have therefore clarified in the Analysis section that, depending on the analysis presented, statistical comparisons were performed either between the two main study periods (pre-pandemic vs post-study resumption) or across the four study periods (P0–P3), as follows (page 11, lines 247-250):

> The quantitative analysis primarily consisted of describing implementation-related indicators across study periods, in line with the RE-AIM framework. Where appropriate, formal statistical comparisons were performed to assess differences either between the two main study periods (pre-pandemic vs post-study resumption) or across the four study periods (P0–P3), depending on the analysis presented.

We have also revised the table and figure legends to specify the statistical comparison underlying each reported P value. No adjustment for multiple comparisons was performed because the analyses were descriptive and exploratory, aiming to characterize implementation outcomes rather than to test multiple prespecified hypotheses.

Results section:

Page 12, lines 278-288: In all, 5,744 pregnant women completed the eligibility questionnaire between March 2017 and April 2023 at the Lille University Hospital maternity ward; 1,804 were deemed eligible for the ECAIL trial. Among the women screened, 29.7% (n=955) were eligible in the pre-pandemic period and 33.6% (n=849) post-resumption (P=0.002, comparison between the two main study periods). The eligibility rate remained consistently higher across each of the three post-resumption subperiods (P1–P3), as illustrated in **Fig 2**.

Fig 2. Rates of eligibility for the ECAIL trial (n=1,804), and rates of in-person delivery of study information (n=915) and participation (n=443) among eligible women according to the four study periods; pre-pandemic, P0: March 2017 to March 2020; post-resumption, P1: September 2020 to August 2021; P2: September 2021 to August 2022; P3: September 2022 to April 2023. *p* value from χ^2 test comparing P0, P1, P2 and P3. The ECAIL trial, Lille study area.

Table 2 footnote (page 14, lines 305-306): p-values compare the pre-pandemic (March 2017–March 2020) and post-resumption (September 2020–April 2023) periods using χ^2 tests.

Page 14, lines 313-314: **Fig 3. Distribution of social vulnerabilities among eligible women (n=1,804) according to the four study periods; pre-pandemic, P0: March 2017 to March 2020; post-resumption, P1: September 2020 to August 2021; P2: September 2021 to August 2022; P3: September 2022 to April 2023. *p* value from χ^2 test comparing P0, P1, P2 and P3. The ECAIL trial, Lille study area.**

Table 3 footnote (page 15, line 329): Within each study period, p-values compare participants and non-participants using χ^2 tests.

Table 4 footnote (page 18, lines 369-370): p-values compare the pre-pandemic (March 2017–March 2020) and post-resumption (September 2020–April 2023) periods using χ^2 tests.

4.14. Lines 193–194. The failed-visit rate uses "number of trips made" as the denominator, which differs from the skipped-visit denominator (theoretical visits). Please report the number of trips per period, since this denominator is not currently shown.

Following Reviewer 1's Comment 1.4, Table 2 now presents percentages only, with the corresponding denominators reported in the table footnotes. To address the present comment, we have also specified the numbers underlying the skipped- and failed-visit indicators, including the numbers of theoretical visits and trips made during each study period, as follows (Table 2 footnote; page 14, lines 306-309):

[a] Follow-up among 443 participants, corresponding to 919 theoretical visits before the pandemic and 1,397 after study resumption. [b] The numbers of skipped visits were 16 before the pandemic and 30 after study resumption. [c] The numbers of failed visits (and corresponding trips made) were 47 (959) before the pandemic and 23 (1,390) after study resumption.

Results

4.15. Line 253 vs Fig 2. The eligibility comparison is reported as P=0.002 in the text and P=0.02 in Fig 2. These appear to reflect a two-period test (text) and a four-period test (figure), consistent with the pattern elsewhere, but this is never stated. Please make the comparison explicit for each P-value so readers are not left to infer that the same comparison yielded two values.

We apologize for the lack of clarity. The p-value reported in the Results section (p = 0.002) corresponds to the comparison between the two main study periods (pre-pandemic vs post-study resumption), whereas Figure 2 reports the p-value from the analysis across the four study periods (P0–P3). These are therefore two

different statistical analyses rather than inconsistent results. To avoid this ambiguity, we have clarified in the Analysis section that statistical comparisons were performed either between the two main study periods or across the four study periods, depending on the analysis presented (please see our response to Comment 4.13). The Results section and the Figure 2 legend were also revised accordingly (please see our response to Comment 4.13).

4.16. Table 2 and Fig 2 caption. The in-person delivery figures are internally inconsistent. Fig 2 reports n=915 in-person deliveries, and Table 2 reports 50.7% under "Screening (n=1789)." However, 915/1789 = 51.1%, whereas 915/1804 (all eligible) = 50.7%. Please reconcile the denominator (1789 vs 1804) and the count/percentage.

We thank the reviewer for identifying this inconsistency. This resulted from a typographical error in Table 2. The table has now been corrected so that the denominator, counts, and percentages are fully consistent with Figure 2 (pages 13-14, Table 2).

Table 2. Distribution of social vulnerabilities and study implementation indicators among eligible women by study period. The ECAIL trial, Lille study area.

	March 2017 to Apr 2023 % (n=1,804)	March 2017 to March 2020 % (n=955)	Sept 2020 to Apr 2023 % (n=849)	*p*-value
**Social vulnerabilities**				
Receives social or medical benefits	78.3	77.2	79.5	0.23
Household's main source of income is not employment	58.7	56.8	60.9	0.07
Perceived financial hardship	24.4	25.8	23.0	0.17
Social isolation	14.7	14.3	15.1	0.66
Precarious housing	6.0	5.5	6.6	0.35
≥ 3 social vulnerabilities	19.0	18.8	19.2	0.81
**Study implementation indicators**				
Screening				
In-person delivery of study information	50.7	45.1	57.0	<0.0001
Follow-up[a]				
Skipped visits[b]	2.0	1.7	2.1	0.49
Failed visits[c]	3.0	4.9	1.7	<0.001

p-values compare the pre-pandemic (March 2017–March 2020) and post-resumption (September 2020–April 2023) periods using χ^2 tests. [a] Follow-up among 443 participants, corresponding to 919 theoretical visits before the pandemic and 1,397 after study resumption. [b] The numbers of skipped visits were 16 before the pandemic and 30 after study resumption. [c] The numbers of failed visits (and corresponding trips made) were 47 (959) before the pandemic and 23 (1,390) after study resumption.

4.17. Line 320. Among ineligible women, "19.1% (n=746)" is reported, but 746 / (5,744 − 1,804 = 3,940) = 18.9%. Please verify the denominator and correct the percentage.

This was another typographical error inadvertently introduced during the final updating of the numbers before manuscript submission and not detected during proofreading. The percentage has now been corrected so that it is fully consistent with the reported numerator and denominator (page 16, line 355).

> Among ineligible women, 18.9% (n=746) were identified as experiencing at least one social vulnerability criterion (according to the eligibility questionnaire) but also met at least one exclusion criterion.

4.18. Fig 4 (n=1345). The number of nonparticipants implied by the data (1,804 eligible − 443 participants = 1,361) does not match the n=1,345 shown. Together with the 443/444 and 1789/1804 discrepancies, this suggests unreported missing data. Please reconcile all denominators and state explicitly how missing or incomplete questionnaires were handled.

This discrepancy also resulted from a typographical error introduced during the final updating of the numbers before manuscript submission and not detected during proofreading. Figure 4 has now been corrected, and all reported denominators are internally consistent (page 16, line 342). We confirm that no participants or questionnaires were excluded from this analysis because of missing data.

Fig 4. Distribution of the four main reasons for nonparticipation in the study among eligible nonparticipating women (n=1,361) by period (pre-pandemic and post-resumption). The ECAIL trial, Lille study area.

4.19. Table 4. The reported P-value for "Serious illness during pregnancy" (17.9% prepandemic vs 18.8% post-resumption, P=0.15) appears inconsistent with the percentages and sample sizes (n=453 and n=293); a χ2 test of these proportions yields a much larger P (≈0.7). Please recheck this value and verify the other P-values in the table.

We thank the reviewer for identifying this additional typographical error. The reported *p*-value has now been corrected. We have also carefully rechecked all the other *p*-values reported in Table 4 and confirm that they are correct (pages 17-18, Table 4).

Table 4. Distribution of inclusion and exclusion criteria among ineligible women facing social disadvantage, by study period. The ECAIL trial, Lille study area.

	March 2017 to Apr 2023 % (n=746)	March 2017 to March 2020 % (n=453)	Sept 2020 to Apr 2023 % (n=293)	*p*-value
**Inclusion criteria: social vulnerabilities**				
Receives social or medical benefits	75.1	77.0	72.0	0.12
Household's main source of income is not employment	65.1	65.3	64.8	0.89
Perceived financial hardship	31.0	31.1	30.7	0.91
Social isolation	17.6	15.0	21.5	0.02
Precarious housing	14.9	11.7	19.8	0.002
≥ 3 social vulnerabilities	27.1	25.8	29.0	0.34
**Exclusion criteria**				
Planning to move outside the study area before the child's first birthday	30.0	34.4	23.2	0.001
Moved several times in the past 12 months	20.0	16.6	25.3	0.004
Sheltered in emergency centers, portable dwellings or homeless	10.3	7.1	15.4	<0.001
Suboptimal understanding/speaking of French	20.6	17.9	24.9	0.02
Serious illness during pregnancy	18.2	17.9	18.8	0.76
Under legal guardianship	5.0	3.8	6.8	0.059
Multiple pregnancy (≥3 fetuses)	1.1	1.5	0.3	0.16
Illegal substance use (excluding cannabis)	2.4	1.8	3.4	0.15

p-values compare the pre-pandemic (March 2017–March 2020) and post-resumption (September 2020–April 2023) periods using χ^2 tests.

4.20. Lines 261–272 and 285–287. Much of the narrative emphasizes four-period trends ("highest during P1," "declined progressively to P3," participation "rose during P1 and P2"). If these subperiod differences were not formally tested, please describe them as descriptive observations rather than findings, and avoid implying statistical significance.

We agree that the description of the subperiod results should not be interpreted as implying formal pairwise comparisons between individual post-resumption periods. Our intention was to describe the observed patterns shown in Figures 2 and 3, while the statistical analyses for these figures were based on overall χ^2 tests across the four study periods (P0–P3). To avoid any ambiguity, we have revised the wording in the

Results section to make it clear that these statements describe the observed distributions across subperiods rather than statistically significant differences between individual periods.

> Page 13, lines 294-301: However, when examined across post-resumption subperiods, **Fig 3** shows that the highest observed proportion of eligible women for whom the main source of income in the household did not derive from employment occurred during P1 (64.8%), as did the receipt of social or medical benefits (82.8%). The highest observed proportion of women reporting financial hardship occurred during P3 (29.3%). Only 50.7% of eligible women received information on the study in person from the ECAIL dietitians, rather than by telephone, but this rate was higher post-resumption (57.0% compared with 45.1% before the pandemic, **Table 2**). **Fig 2** shows that the highest observed rate occurred during P1 (59.6%) before decreasing to 51.7% in P3.

> Page 14, lines 318-320: **Fig 2** shows that participation was highest during P1 (26.9%) and P2 (28.7%) and lower during P3 (20.1%), falling below the pre-pandemic level observed in P0 (23.4%).

4.21. Lines 337–339 and 359–360. The drop in failed visits (4.9% to 1.7%) is interpreted as an effect of the pre-visit questionnaire. Because the pre-visit call confirms presence before a trip is made, fewer wasted trips is partly a mechanical consequence of the procedure rather than an independent outcome; please present this more cautiously.

We agree that the reduction in failed visits should not be interpreted as an independent effect of the pre-visit questionnaire. Rather, the questionnaire functioned as an implementation adaptation that allowed dietitians to identify participant unavailability before travelling, thereby avoiding unnecessary trips and saving time. We have revised the wording of the qualitative findings to better reflect this mechanism (page 19, lines 398-400), while retaining the quantitative description of the observed reduction in failed visits.

> Finally, the pre-visit COVID-19 risk assessment questionnaire was perceived as an effective implementation adaptation, helping to avoid unnecessary trips when participants were unavailable and thereby saving time.

4.22. Table 3. For precarious housing, the direction of association reverses between periods (participants higher prepandemic, 7.2% vs 5.1%; participants lower post-resumption, 3.2% vs 7.8%, P=0.02). This is worth a sentence of interpretation, as it runs counter to the broader narrative of increasing precarity.

We agree that this finding deserves to be highlighted. We have therefore added a sentence in the Results section noting that precarious housing was the only social vulnerability indicator for which the direction of the association with participation differed between the pre-pandemic and post-resumption periods (page 14, lines 323-325). Given the small number of women concerned and the descriptive nature of these analyses, we chose not to provide a speculative interpretation.

> One exception concerned precarious housing, for which the direction of the association with participation differed between the pre-pandemic and post-resumption periods.

Discussion

4.23. Lines 366–378. The opening synthesis attributes the expansion of the eligible pool and sustained engagement to the contextual shocks. Given the design, please frame these as plausible interpretations and explicitly acknowledge that secular trends, changes in screening/prescreening and referral practices over six years, team maturation, and other concurrent changes cannot be ruled out as contributors.

We agree that the observational design precludes causal attribution. In response to this and related comments from the reviewers, we have substantially revised the opening paragraph of the Discussion to adopt more cautious language throughout. The revised text now consistently refers to associations and plausible interpretations (e.g., "suggest", "were associated with", "may have reinforced"), rather than causal effects (pages 20-21, lines 406-418).

> This study of an interdisciplinary public health intervention among socially disadvantaged families provides implementation-relevant insights into how trial implementation evolved across the COVID-19 pandemic and the

> subsequent cost-of-living crisis. Interpreted through a RE-AIM lens (19, 20), our findings suggest that these contextual shocks were associated with an increase in the proportion of women meeting the trial eligibility criteria, without compromising provider *Adoption* or *Implementation*, and may even have reinforced engagement during key phases of delivery. Quantitative and qualitative findings converged, showing shifts in eligibility and participation profiles alongside sustained involvement of both implementers and participants. This resulted in a stable rhythm of home visits after resumption, with some parents eager to re-establish social contact, suggesting that home visits may have provided benefits extending beyond intervention delivery. These health and economic shocks also affected socially disadvantaged families not eligible for the ECAIL trial, exacerbating existing social and material vulnerabilities. Taken together, these findings suggest overall robust implementation despite major contextual disruptions and may inform the design of more resilient interventions.

Regarding alternative explanations, we acknowledge that observational data cannot exclude the contribution of other contextual changes over time. However, several potential sources of bias raised by the reviewer, including changes in prescreening procedures and referral practices, are unlikely explanations in the present study, as these procedures remained unchanged throughout the study period and have been clarified in the Methods and response to Comment 4.8. We therefore chose not to expand the Discussion with speculative explanations that are not supported by our data.

4.24. Lines 403–407. The suggestion that reduced perceived financial hardship during P1 reflects households "accumulating savings" during lockdown rests on a national average (consumption fell 7.1%) and is questionable for a low-income, socially vulnerable population, for whom the benefit increases described at lines 401–402 are a more plausible explanation. Please reconsider or temper this mechanism.

We agree that the increase in social and medical benefit receipt provides the most direct explanation for the lower prevalence of perceived financial hardship during P1. We have therefore revised this paragraph to give greater emphasis to this explanation and tempered the alternative mechanism related to reduced household spending during lockdowns. We no longer refer to the accumulation of savings and instead state more cautiously that temporary reductions in household spending may also have contributed to reducing short-term financial pressures for some households (page 21, lines 437-441).

> The higher proportion of ECAIL-eligible women receiving social or medical benefits during P1 is consistent with national administrative data showing a sharp increase in benefit receipt early in the pandemic (39). This may help explain the concurrent decrease in perceived financial difficulties. Temporary reductions in household spending during lockdowns, when consumption expenditure fell markedly (40), may also have played a role in reducing short-term financial pressures for some households.

4.25. Lines 455–460. "this investment appears cost-effective" is not supported, as no cost or cost-effectiveness data were collected. Please soften to efficiency in terms of time and wasted trips, or remove the cost-effectiveness claim.

We agree that the term "cost-effective" was too strong, as no formal economic evaluation was conducted. We have therefore replaced it with a more appropriate formulation referring to the efficiency of trial implementation. This wording better reflects our findings, which indicate that the pre-visit procedures reduced unnecessary trips, facilitated visit rescheduling, and improved the use of staff time, without implying a formal cost-effectiveness evaluation (page 34, lines 495-498).

> However, this investment may improve the efficiency of trial implementation by reducing unnecessary trips and facilitating visit rescheduling, thus saving time and subsequent manhour costs in the long term, while also enhancing adherence to the study protocol, participant commitment, and data quality by limiting missed visits.

4.26. Throughout the Discussion and Conclusion. Provider engagement (supported by the qualitative data and the in-person delivery indicator) and participant engagement/reach (mixed: stable participation, declining in P3) are at times conflated under "engagement." Please distinguish these clearly, since the evidence differs in strength.

We agree that provider engagement and participant engagement should be more clearly distinguished throughout the manuscript. We have therefore reviewed the Discussion and Conclusion and revised the wording where necessary to explicitly specify whether statements referred to provider engagement (e.g., HCP engagement and implementation processes) or participant recruitment and engagement. These revisions ensure that the interpretation of the findings remains aligned with the corresponding quantitative and qualitative evidence (lines 101, 133, 196, 522).

4.27. Lines 376–378 and 481–482. Claims of "robust implementation" should be reconciled with the P3 decline in participation and in-person delivery. The data arguably show stability with weakening in later periods, which is a more nuanced picture than uniform robustness.

We agree that the findings are more nuanced than uniformly robust implementation across all study periods. We have therefore slightly tempered the wording of the opening paragraph of the Discussion by referring to *overall* robust implementation rather than robust implementation, which better reflects the balance of the findings while acknowledging that some implementation indicators weakened during the final study period.

> Pages 20-21, lines 416-418: Taken together, these findings suggest overall robust implementation despite major contextual disruptions and may inform the design of more resilient interventions.

4.28. Lines 381–423. This section relies heavily on external Insee/Ipsos statistics to build a mechanistic narrative. While useful for context, the study cannot test these mechanisms; consider trimming and signposting more clearly which statements are study findings versus external context.

We agree that national statistics should be used to provide contextual interpretation rather than to infer mechanisms directly from our data. In response to comments from the reviewers, we substantially shortened this section of the Discussion and revised the wording throughout to more clearly distinguish the findings of the present study from contextual information derived from national sources. The external data are now used solely to contextualize our observations and to support plausible interpretations, using appropriately cautious language (e.g., "consistent with", "suggest", "may explain", "may also have played a role"), rather than to imply that these mechanisms were directly demonstrated by our study (pages 20-21, lines 421-457).

> **Eligibility, ineligibility, and social vulnerabilities**
> The consistently high eligibility rate observed in the ECAIL trial, even before the pandemic, is consistent with the well-documented socioeconomic disadvantage of the Hauts-de-France region, shaped by the economic decline following the collapse of its coal, textile, and steel industries in the 1980s (30). In 2020, 17.2% of the population lived below the poverty line, compared with 13.6% nationally (24, 31). The *Nord* district, where Lille is located, also has the highest poverty rate in the region (18.4%) (24), together with higher levels of unemployment (32), lower educational attainment (33, 34), a greater proportion of blue-collar workers (33, 35), and more single-parent families (36, 37).
> Against this already disadvantaged socioeconomic backdrop, the higher post-resumption eligibility rate observed in ECAIL suggests that the COVID-19 pandemic further exacerbated existing inequalities. This interpretation is consistent with national evidence showing that low-income households were disproportionately affected by labor-market disruptions and were less likely to hold stable jobs compatible with teleworking (38). As a result, household living standards declined. Labor-market disruptions, including those affecting lower middle-class households, likely contributed to the higher post-resumption eligibility rate, consistent with the increased proportion of women reporting that employment was not their household's main source of income during P1. Likewise, at the national level, the poverty rate increased from 13.6% in 2020 to 15.4% in 2023 (31).
> The higher proportion of ECAIL-eligible women receiving social or medical benefits during P1 is consistent with national administrative data showing a sharp increase in benefit receipt early in the pandemic (39). This may help explain the concurrent decrease in perceived financial difficulties. Temporary reductions in household spending during lockdowns, when consumption expenditure fell markedly (40), may also have played a role in reducing short-term financial pressures for some households.
> The persistently high eligibility rate nonetheless observed during P2 and P3 suggests that the socioeconomic consequences of the pandemic extended well beyond the acute health crisis, likely reflecting persistent macro-economic pressures, including rising energy and food prices. Although employment partially recovered after restrictions were lifted, inflation and the withdrawal of exceptional government financial support introduced during the pandemic likely contributed to the renewed increase in reported financial difficulties during P3. More broadly,

the cost-of-living crisis compounded the challenges faced by a population already weakened by the pandemic. This interpretation is consistent with national surveys documenting worsening financial hardship and food insecurity during this period (41). This worsening socioeconomic context is also reflected in our analysis of women ineligible for the ECAIL trial despite experiencing social vulnerability. These women faced increasing housing precarity and instability, as well as heightened social isolation, consistent with previous findings from the Haut-de-France region (42). These findings also suggest that the minimum level of stability required to participate in longitudinal intervention studies may inadvertently exclude some women experiencing social vulnerability, an issue that warrants further investigation. Future research should explore strategies to reduce participation burden and develop implementation approaches that better reach families experiencing the highest levels of social disadvantage.
Taken together, these results underscore how multidimensional crises such as the COVID-19 pandemic can intensify pre-existing vulnerabilities, highlighting the need for resilient intervention designs.

4.29. Lines 413–418. The food-insecurity figure (fresh fruit/vegetables, 27% in 2018 to 43% in 2023) lacks a citation; please add the source.

The original version of the manuscript included an appropriate citation (formerly Ref. 41) supporting this statement. During the revision process, however, the sentence referring specifically to the increase in food insecurity was removed as part of a broader effort to streamline the Discussion. The revised text now presents this point more concisely while retaining the appropriate reference to national survey data documenting worsening financial hardship and food insecurity during this period (page 22, lines 448-449).

This interpretation is consistent with national surveys documenting worsening financial hardship and food insecurity during this period (41).

Limitations and strengths

4.30. Lines 475–492. The limitations are currently confined mainly to recall bias. Please expand to address: (a) the before/after design without a control group and the resulting inability to attribute changes to the crises rather than to secular or implementation-related trends; (b) the single-site focus (Lille only; Valenciennes excluded), limiting generalizability; (c) the dependence of the eligibility rate on prescreening/distribution practices; (d) multiple testing without adjustment; (e) qualitative analysis by a single coder and the secondhand nature of participants' reported views; and (f) unreported missing data and the denominator inconsistencies noted above.

We agree that the limitations section should more explicitly acknowledge the main methodological constraints of the study. We have therefore expanded this section to discuss the observational before-and-after design without a control group, which precludes causal attribution, and the restriction of the quantitative analyses to the Lille study site, which may limit the generalizability of the findings (pages 24-25, lines 517-544).

Limitations and strengths
One limitation of this study is that the qualitative component relied exclusively on a focus group with dietitians, whose perspectives may not fully reflect those of other stakeholders involved in trial implementation. Participation in the focus group was also unequal, with two dietitians contributing more extensively than the others, although quotations were selected to illustrate the identified themes rather than to reflect the relative frequency of individual contributions. Because the focus group was conducted two years after the last lockdown, recall bias cannot be excluded. Moreover, the before-and-after observational design without a control group precludes attributing the observed changes specifically to the COVID-19 pandemic and the cost-of-living crisis, as other temporal influences cannot be excluded. Finally, because the quantitative analyses were restricted to the Lille site, where recruitment started before the pandemic, the findings may not be fully generalizable to other implementation settings. Nonetheless, the vividness of the dietitians' recollections underscores the lasting impact of this period and the relevance of capturing these experiences retrospectively. Although effectiveness outcomes are not examined here, limiting conclusions about whether successful implementation translated into intervention benefit, this focus is consistent with the RE-AIM framework's emphasis on disentangling *reach*, *adoption*, and *implementation* processes as necessary precursors to effectiveness and sustainability (19). A major strength lies in the triangulation of quantitative and qualitative methods (28, 44, 47), which enabled a richer understanding of both the evolving

> characteristics of the target population and the implementation processes of the intervention across successive health and economic crises. Importantly, the inclusion of dietitians' perspectives represents a further strength because they were the frontline implementers of the trial and directly involved in participant recruitment, follow-up and data collection. Their perspectives, which are still relatively rarely captured in implementation research, provided valuable insights into day-to-day implementation during the successive crises. The study's focus on pregnant women experiencing social vulnerabilities, a heterogeneous population that remains largely underrepresented in epidemiological research, provides new knowledge about the specific barriers to intervention delivery and participant engagement in a context of worsening poverty. The adjustments made after the onset of the COVID-19 pandemic illustrate the flexibility of this complex interventional research in responding to unforeseen events and adapting implementation to enable study continuation (46, 48). They also suggest that home visits and one-to-one interactions may strengthen participation and commitment, despite constraints on face-to-face contact.

Several other points raised by the reviewer have also been addressed elsewhere in the revised manuscript or in response to previous comments. Specifically, we clarified the stability of the prescreening procedures (comment 4.8), the qualitative coding procedures and independent review of themes (comment 4.12), the rationale for not adjusting for multiple comparisons given the descriptive implementation objectives of the study (comment 4.13), and corrected the denominator inconsistencies identified in the tables (comments 4.2 and 4.14-20). We therefore considered that repeating these points in the Limitations section would be unnecessarily redundant.

4.31. Lines 478–481. The absence of Effectiveness and Maintenance outcomes is framed as consistent with RE-AIM's staged logic. This is reasonable, but it remains a limitation for conclusions about whether reach/engagement translated into benefit; please present it as such.

We agree that the absence of effectiveness and longer-term maintenance outcomes should also be acknowledged as a limitation of the present study. We have therefore revised this sentence to explicitly state that, although the focus on *Reach*, *Adoption*, and *Implementation* is consistent with the RE-AIM framework, it limits conclusions about whether successful implementation translated into intervention effectiveness or longer-term benefit (page 25, lines 528-531).

> Although effectiveness outcomes are not examined here, limiting conclusions about whether successful implementation translated into intervention benefit, this focus is consistent with the RE-AIM framework's emphasis on disentangling *reach*, *adoption*, and *implementation* processes as necessary precursors to effectiveness and sustainability (19).

Conclusions

4.32. Lines 494–508. The conclusion is somewhat more optimistic than the data support and largely restates the Discussion. Please temper the claims of resilience to reflect the temporal heterogeneity (improvement in P1, decline by P3), and ensure conclusions about "engagement" specify provider versus participant engagement.

We agree that the conclusion should reflect the overall pattern of findings without overstating resilience across all study periods. In response to this and related comments, we have further tempered the concluding statements by using more cautious wording regarding intervention resilience. We also reviewed the Conclusion to ensure that provider engagement and participant engagement are clearly distinguished throughout, and slightly streamlined the text to avoid unnecessary repetition of points already developed in the Discussion. We believe the revised Conclusion more accurately reflects the temporal heterogeneity observed across the study while preserving the main implementation message (page 26, lines 547-559).

> The social impact of both the pandemic and the cost-of-living crisis, often described as the "second health emergency" (5), was associated with a higher eligibility rate among socially disadvantaged families in the ECAIL trial, while participation remained stable. These findings provide important insights into how successive crises may influence participation in programs designed to mitigate social disadvantage. In the ECAIL trial, the collaborative foundations of this co-created intervention may have helped sustain both stakeholder and participant engagement

despite increased workload and organizational strain experienced within hospitals during the successive crises. Moreover, home visits and telephone calls between follow-ups were perceived as effective implementation strategies, not only for reaching families and supporting adherence, but also for mitigating social isolation among participants. Overall, these findings suggest that interventions co-designed with stakeholders and target populations, and supported by appropriate implementation strategies, may remain broadly resilient during major external disruptions, highlighting the value of flexible, context-sensitive implementation approaches in a context of rising poverty and ongoing geopolitical and economic instability.
