# Supplementary material for: Reach and engagement in the ECAIL trial targeting socially disadvantaged families across the COVID-19 pandemic and cost-of-living crisis: A mixed-methods implementation study
Source: PLoS One. 2026 Oct 8;21(10):e0350372. doi: 10.1371/journal.pone.0350372 (PMC13649008; doi:10.1371/journal.pone.0350372)
Supplement: Peer review history

# Peer review: Decision Letter 0

**DOI:** 10.1371/journal.pone.0350372.r001

**Authors:** Javier Fagundo-Rivera

29 Jun 2026

Dear Dr. Lioret,

Thank you for submitting your manuscript to PLOS ONE. After careful consideration, we feel that it has merit but does not fully meet PLOS ONE’s publication criteria as it currently stands. Therefore, we invite you to submit a revised version of the manuscript that addresses the points raised during the review process.

==============================

**ACADEMIC EDITOR:**

Dear Authors,

Thank you for submitting your manuscript entitled “ How the COVID-19 pandemic and cost-of-living crisis shaped reach and engagement in the ECAIL trial targeting socially disadvantaged families: an interdisciplinary implementation study ” (Manuscript Number: PONE-D-26-23207) to Plos One.

After evaluation by four independent reviewers and editorial assessment, the current decision is Major Revision.

The reviewers recognize the potential relevance and originality of the topic. However, they also identified several important issues that should be addressed before the manuscript can be considered further for publication. These concerns include aspects related to methodological clarification, interpretation of findings, statistical reporting, and improvement of the overall presentation and discussion of the results.

Please revise the manuscript carefully by addressing each reviewer comment point-by-point in a detailed response letter. We encourage you to clearly indicate all modifications made in the revised manuscript, preferably using track changes or highlighted text.

We look forward to receiving your revised submission and appreciate your contribution to the field.

Sincerely,

Javier Fagundo-Rivera, PhD

Academic Editor

==============================

Please submit your revised manuscript by Aug 28 2026 11:59PM. If you will need more time than this to complete your revisions, please reply to this message or contact the journal office at plosone@plos.org. When you're ready to submit your revision, log on to https://www.editorialmanager.com/pone/ and select the 'Submissions Needing Revision' folder to locate your manuscript file.

- A letter that responds to each point raised by the academic editor and reviewer(s). You should upload this letter as a separate file labeled 'Response to Reviewers'.
- A marked-up copy of your manuscript that highlights changes made to the original version. You should upload this as a separate file labeled 'Revised Manuscript with Track Changes'.
- An unmarked version of your revised paper without tracked changes. You should upload this as a separate file labeled 'Manuscript'.

If you would like to make changes to your financial disclosure, please include your updated statement in your cover letter. Guidelines for resubmitting your figure files are available below the reviewer comments at the end of this letter.

If applicable, we recommend that you deposit your laboratory protocols in protocols.io to enhance the reproducibility of your results. Protocols.io assigns your protocol its own identifier (DOI) so that it can be cited independently in the future. For instructions see: https://journals.plos.org/plosone/s/submission-guidelines#loc-laboratory-protocols. Additionally, PLOS ONE offers an option for publishing peer-reviewed Lab Protocol articles, which describe protocols hosted on protocols.io. Read more information on sharing protocols at https://plos.org/protocols?utm_medium=editorial-email&utm_source=authorletters&utm_campaign=protocols.

As the corresponding author, your ORCID iD is verified in the submission system and will appear in the published article. PLOS supports the use of ORCID, and we encourage all coauthors to register for an ORCID iD and use it as well. Please encourage your coauthors to verify their ORCID iD within the submission system before final acceptance, as unverified ORCID iDs will not appear in the published article. *Only* the individual author can complete the verification step; PLOS staff *cannot* verify ORCID iDs on behalf of authors.

We look forward to receiving your revised manuscript.

Kind regards,

Javier Fagundo-Rivera, PhD

Academic Editor

PLOS One

Journal Requirements:

When submitting your revision, we need you to address these additional requirements.

1.Please ensure that your manuscript meets PLOS ONE's style requirements, including those for file naming. The PLOS ONE style templates can be found at

https://journals.plos.org/plosone/s/file?id=wjVg/PLOSOne_formatting_sample_main_body.pdf and

https://journals.plos.org/plosone/s/file?id=ba62/PLOSOne_formatting_sample_title_authors_affiliations.pdf

2. Please note that funding information should not appear in any section or other areas of your manuscript. We will only publish funding information present in the Funding Statement section of the online submission form. Please remove any funding-related text from the manuscript.

3. Please include your full ethics statement in the ‘Methods’ section of your manuscript file. In your statement, please include the full name of the IRB or ethics committee who approved or waived your study, as well as whether or not you obtained informed written or verbal consent. If consent was waived for your study, please include this information in your statement as well.

4. Please include captions for your Supporting Information files at the end of your manuscript, and update any in-text citations to match accordingly. Please see our Supporting Information guidelines for more information: http://journals.plos.org/plosone/s/supporting-information.

5. If the reviewer comments include a recommendation to cite specific previously published works, please review and evaluate these publications to determine whether they are relevant and should be cited. There is no requirement to cite these works unless the editor has indicated otherwise.

**Additional Editor Comments:**

Dear Authors,

Thank you for submitting your manuscript entitled “ How the COVID-19 pandemic and cost-of-living crisis shaped reach and engagement in the ECAIL trial targeting socially disadvantaged families: an interdisciplinary implementation study ” (Manuscript Number: PONE-D-26-23207) to Plos One.

After evaluation by four independent reviewers and editorial assessment, the current decision is Major Revision.

The reviewers recognize the potential relevance and originality of the topic. However, they also identified several important issues that should be addressed before the manuscript can be considered further for publication. These concerns include aspects related to methodological clarification, interpretation of findings, statistical reporting, and improvement of the overall presentation and discussion of the results.

Please revise the manuscript carefully by addressing each reviewer comment point-by-point in a detailed response letter. We encourage you to clearly indicate all modifications made in the revised manuscript, preferably using track changes or highlighted text.

We look forward to receiving your revised submission and appreciate your contribution to the field.

Sincerely,

Javier Fagundo-Rivera, PhD

Academic Editor

[Note: HTML markup is below. Please do not edit.]

Reviewer's Responses to Questions

**Comments to the Author**

1. Is the manuscript technically sound, and do the data support the conclusions?

Reviewer #1: Partly

Reviewer #2: Yes

Reviewer #3: Yes

Reviewer #4: Yes

**********

2. Has the statistical analysis been performed appropriately and rigorously?

Reviewer #1: No

Reviewer #2: Yes

Reviewer #3: Yes

Reviewer #4: Yes

**********

3. Have the authors made all data underlying the findings in their manuscript fully available?

The PLOS Data policy

Reviewer #1: No

Reviewer #2: Yes

Reviewer #3: Yes

Reviewer #4: Yes

**********

4. Is the manuscript presented in an intelligible fashion and written in standard English?

Reviewer #1: Yes

Reviewer #2: Yes

Reviewer #3: Yes

Reviewer #4: Yes

**********

**Reviewer #1:**

One of the best-written introductions I've ever reviewed, but the rest of the submission is poorly organized and I'm not sure whether the study is sound because it loses its cohesion. If this study is mixed methods, the methods must inform one another, demonstrate how the quantitative data affects the qualitative analysis (or vice versa - ideally both) and there must be some sort of plan or citation (ideally both) for how to go about developing interconnected analysis. What is here at present is a lot of descriptive statistics without significance, followed by a rushed qualitative report (I hesitate to call it thematic since there is no theory behind how themes are identified or reported) on a focus group where 2/6 participants deliver 90% of the quotations.

One of the fundamental flaws is that while multiple time periods are introduced and compared, no information is given about how these time periods might vary in the healthcare setting. There are brief mentions of multiple lockdowns, masking standards, and relaxation of mitigations, but we never see these laid out, let alone conferred with the timeline of data collection and patient engagement. What conventions changed over these four periods? Don't assume that COVID was the same for all readers - walk them through what it was like in Lille. Contextualize it with brief comparisons to other cities, the rest of France and/or Europe, the rest of the world.

Discussion section introduces far too many new topics to an already-dense study; authors shouldn't have to cite much by this point, it should all be part of the introduction, methods, and data sections, then outlined in analysis and reported in results, and then under discussion nothing is left except to reflect on implications of findings and future directions.

Tables are sloppy, and much of the information shown there could be relegated to supplemental materials. Tables 2, 3, and 4: Please indicate percentage in the headers of relevant columns rather than the table header. I'm not always clear which column these p-values (note the lowercase p) report for. Also, please indicate items where p-value is statistically significant with an asterisk (*). Table 5 is dominated by only Dietitians 1 and 2; some of the quotations are even redundant.

Supplemental documents are attached but not introduced/discussed. Standardized questionnaires can just be introduced and cited, but they must be introduced.

238-242: It is not standard to have thematic coding performed by a single researcher, especially for a team this size. The circumstances appear unique enough and the scope narrow enough that it doesn't endanger the paper, however if any other researchers or participants even briefly reviewed the content analysis (see 669-671), I strongly recommend elaborating it here.

295-298 and Figure 4: I think this can be reported in text without giving an entire page to a diagram lacking significance.

319-330 This paragraph is an insightful reminder that even among "disadvantaged" patients, the minimum stability necessary to participate can and does exclude patients who are more disadvantaged. This section highlights an opening for future research on two topics: addressing the demands placed on patients who participate in research and addressing increases in social disadvantage in the years immediately following COVID lockdowns.

337 no *significant difference

346-348: at no point are these "remote solutions" introduced or discussed, let alone dieticians' reasons for rejecting them.

353: where was ECAIL prioritized before resumption? what else was it competing with for clinical attention? Table 5 includes a quotation implying that a lot of other programs were still on pause when ECAIL resumed - this link should be explored in discussion and possibly earlier.

354-359: say more about the impact on social isolation (perhaps in discussion) and mask-wearing (protocols, tensions, and timing of relaxation of masking standards), and ideally confer these conditions against contemporary research (about social isolation and masking, respectively).

359-360: what was the risk assessment questionnaire, how was it implemented, and did its implementation change before the end of the trial?

Discussion: why was in-person delivery of information more common after lockdown than before? any theories?

366-371: This overstates the authors' case; the present study may contribute original research on these topics but it is far from proven.

380-400: Discussion is too late to introduce this profile of the community involved; it should go much earlier, under "study design and participants".

405-407: not relevant

411-413: authors have not reported when and how health restrictions were lifted, nor defined "exceptional support measures", see also 476-477.

419-423: say more

427-4460: this section seems heavy on analysis, which should come before discussion but after and separate from Methods. It also elaborates on relevant topics that were not established under Methods (such as concerns over masking).

434-435: the favorable conditions are demonstrated, but not the equity-oriented approach. Authors must introduce this approach (background, importance, relevance to stakeholders) much earlier.

437-446: repetitive of earlier sections, though this is the more appropriate place for it.

448: In my field, we usually call this "recommendations"

455-463: this passage conveys the most significant contribution to research, and it feels buried under everything else. What can be trimmed to give this point more room for discussion?

465-473: messy and unhelpful

476: was it a group interview or a focus group? Be consistent with terms. Also, this delay should be mentioned above, under qualitative analysis.

669 and elsewhere: authors keep saying "interdisciplinary" when I think they mean "mixed method", i.e. qualitative and quantitative analysis. "Interdisciplinary" may also apply (if not only your team comprises researchers from multiple fields, but also your approaches to patients and data), but that is not evident in this draft.

**Reviewer #2:**

I appreciate the opportunity to review this study, which addresses a highly relevant and timely topic. Numerous research projects experienced interruptions or modifications due to the COVID-19 pandemic; however, this work stands out for having successfully maintained the implementation of the intervention and documented its effects across different time periods. Among its main strengths are the availability of data collected before, during, and after the pandemic, as well as the integration of quantitative and qualitative methods, providing a broader understanding of implementation processes.

With the aim of contributing to the strengthening of the manuscript, I offer the following comments:

Methodology

- The quantitative findings could be strengthened through multivariable analyses. The reported associations are based primarily on chi-square tests, which limits the ability to evaluate the independent contribution of the different variables examined.

- There is a possible discrepancy in the operationalization of the Adoption dimension of the RE-AIM framework. Traditionally, this dimension refers to the extent to which organizations, institutions, or professionals adopt an intervention. In the present study, adoption appears to be assessed mainly through the mode of delivery of study information to participants. It would therefore be helpful to provide a stronger justification for this methodological decision or reconsider its classification within the conceptual framework.

- There appears to be a potential conceptual overlap between the inclusion criterion of “precarious housing” and the exclusion criteria related to homelessness, emergency accommodation, hotel housing, or housing instability. As currently presented, some participants could potentially meet both inclusion and exclusion criteria simultaneously. The authors should clarify the operational definitions used during the screening process and explain how potentially ambiguous situations were classified.

Results

- Regarding the statement that "contextual shocks expanded the pool of eligible individuals," although the observed difference in eligibility reached statistical significance, the absolute increase was approximately four percentage points. It would be advisable to complement the presentation of results with absolute differences to facilitate assessment of their practical significance.

- It would also be useful to include basic sociodemographic characteristics of the study population, such as age, marital status, educational level, or other relevant variables, as these would contribute to a better characterization of the participants and provide additional context for interpreting the findings.

Discussion

- I suggest explicitly acknowledging, among the study limitations, the potential bias arising from the fact that the qualitative component was based exclusively on a focus group of dietitians. The inclusion of perspectives from other stakeholders, such as participants or clinical staff, could have enriched the understanding of implementation processes.

Conclusions

- It is recommended that the authors moderate statements that imply causal relationships between the pandemic and the observed changes in eligibility or participation. Given that this is an observational and descriptive study, conclusions should be framed primarily in terms of temporal associations rather than causal effects.

I hope these comments are helpful to the authors in revising and strengthening the manuscript.

**Reviewer #3:**

It is not entirely clear to me what is the question this paper is trying to answer and why is it so long. The same information (more people were eligible to participate in this intervention after rather than before the pandemic) could have been more succinctly presented in a letter to the editor type format. It would be better if this paper asked some questions that the authors were answering. As it stands it's more like a progress report of an ongoing study.

**Reviewer #4:**

Abstract

1. Line 32–34 and title. The framing that the pandemic and cost-of-living crisis “shaped” reach and engagement implies causation. The design is a single-site before/after comparison without a control group, so it can describe associations between time periods and outcomes but cannot attribute change specifically to these crises. Consider associational language here and throughout (e.g., “changes in reach and engagement observed across the pandemic and cost-of-living period”).

2. Line 44. Participation is reported as n=443 here and in the main text (lines 115, 257, 284), but Table 2 reports follow-up n=444. Please reconcile and state the enrolled N consistently.

3. Line 46–47. “the introduction of a pre-visit COVID-19 questionnaire reduced missed appointments.” Because the pre-visit telephone call works by confirming presence before a trip is made, a reduction in wasted trips is partly definitional rather than an independent effect; please phrase more cautiously (see Results comment 7).

4. The abstract notes that eligibility increased significantly but does not note that the distribution of vulnerability indicators among eligible women did not change. This distinction matters for interpretation (more eligible women, but not a more vulnerable eligible group); consider making it explicit.

Introduction

1. Lines 65–79. Well-referenced but long on general macroeconomic context. Consider tightening to foreground the specific gap (implementation outcomes under crisis for hard-to-reach groups), which is the genuine contribution.

2. Lines 86–90. Please state up front that only Reach, Adoption, and Implementation are assessed (not Effectiveness or Maintenance), so readers calibrate expectations early.

3. Lines 91–100. The aims are clear, but Aim (ii) again uses causal phrasing (“shaped implementation”); consider aligning with the descriptive design.

Methods

1. Lines 180–184. The eligibility rate denominator is the number of questionnaires completed and returned to dietitians. This denominator is itself shaped by the prescreening phase (lines 135–137) and by reception/HCP behavior, both of which may have changed over six years. If prescreening became more targeted after resumption, the eligibility rate would rise independently of any real change in the underlying population. Please report the number of questionnaires distributed and returned per period, and discuss how changes in prescreening/distribution could affect the eligibility comparison.

2. Lines 175–178. The RE-AIM operationalization needs sharpening. “Delivery modalities” are mapped to both Adoption and Implementation, which is confusing, and mapping in-person versus telephone delivery onto Adoption is non-standard (Adoption usually refers to setting/staff uptake). Please justify the mapping and avoid assigning one indicator to two dimensions. Relatedly, the representativeness comparisons (participants vs nonparticipants vs ineligible on vulnerability) are arguably the strongest Reach contribution and could be framed explicitly as such.

3. Lines 168–172 and Fig 1. Please state explicitly that the suspension window (March–September 2020) is excluded from both periods. More importantly, Fig 1 shows that P1 (Sept 2020–Aug 2021) contains two further lockdown periods (Oct–Dec 2020 and Apr–Jun 2021), so P1 is not a clean “post-resumption” period; this complicates interpreting P1 as early recovery and should be addressed.

4. Lines 182–184. Using in-person delivery as a proxy for HCP engagement is reasonable but indirect; the decline from P1 to P3 could also reflect dietitian availability, staffing, or scheduling. Please acknowledge these alternatives where the indicator is interpreted.

5. Lines 204–242. Several qualitative reporting elements are missing. Transcription, coding, and thematic analysis appear to have been done by a single researcher (DP); please state whether any second coding, peer debriefing, or reflexivity procedures were used. Because participants' reactions are reported secondhand by dietitians, please make clear (here and in Results/Discussion) that no participant data were collected directly in this component. A COREQ (or SRQR) checklist should accompany STROBE given the focus-group component.

6. Lines 233 and 245–246. The manuscript appears to report two-period χ2 tests in the tables/text and four-period χ2 tests in the figures (e.g., the same indicators carry different P-values in Table 2 vs Fig 3). Please state this explicitly in the Analysis section, label each table and figure with the comparison performed, and indicate whether any adjustment for multiple comparisons was made given the number of tests across Tables 2–4 and Figs 2–6.

7. Lines 193–194. The failed-visit rate uses “number of trips made” as the denominator, which differs from the skipped-visit denominator (theoretical visits). Please report the number of trips per period, since this denominator is not currently shown.

Results

1. Line 253 vs Fig 2. The eligibility comparison is reported as P=0.002 in the text and P=0.02 in Fig 2. These appear to reflect a two-period test (text) and a four-period test (figure), consistent with the pattern elsewhere, but this is never stated. Please make the comparison explicit for each P-value so readers are not left to infer that the same comparison yielded two values.

2. Table 2 and Fig 2 caption. The in-person delivery figures are internally inconsistent. Fig 2 reports n=915 in-person deliveries, and Table 2 reports 50.7% under “Screening (n=1789).” However, 915/1789 = 51.1%, whereas 915/1804 (all eligible) = 50.7%. Please reconcile the denominator (1789 vs 1804) and the count/percentage.

3. Line 320. Among ineligible women, “19.1% (n=746)” is reported, but 746 / (5,744 − 1,804 = 3,940) = 18.9%. Please verify the denominator and correct the percentage.

4. Fig 4 (n=1345). The number of nonparticipants implied by the data (1,804 eligible − 443 participants = 1,361) does not match the n=1,345 shown. Together with the 443/444 and 1789/1804 discrepancies, this suggests unreported missing data. Please reconcile all denominators and state explicitly how missing or incomplete questionnaires were handled.

5. Table 4. The reported P-value for “Serious illness during pregnancy” (17.9% prepandemic vs 18.8% post-resumption, P=0.15) appears inconsistent with the percentages and sample sizes (n=453 and n=293); a χ2 test of these proportions yields a much larger P (≈0.7). Please recheck this value and verify the other P-values in the table.

6. Lines 261–272 and 285–287. Much of the narrative emphasizes four-period trends (“highest during P1,” “declined progressively to P3,” participation “rose during P1 and P2”). If these subperiod differences were not formally tested, please describe them as descriptive observations rather than findings, and avoid implying statistical significance.

7. Lines 337–339 and 359–360. The drop in failed visits (4.9% to 1.7%) is interpreted as an effect of the pre-visit questionnaire. Because the pre-visit call confirms presence before a trip is made, fewer wasted trips is partly a mechanical consequence of the procedure rather than an independent outcome; please present this more cautiously.

8. Table 3. For precarious housing, the direction of association reverses between periods (participants higher prepandemic, 7.2% vs 5.1%; participants lower post-resumption, 3.2% vs 7.8%, P=0.02). This is worth a sentence of interpretation, as it runs counter to the broader narrative of increasing precarity.

Discussion

1. Lines 366–378. The opening synthesis attributes the expansion of the eligible pool and sustained engagement to the contextual shocks. Given the design, please frame these as plausible interpretations and explicitly acknowledge that secular trends, changes in screening/prescreening and referral practices over six years, team maturation, and other concurrent changes cannot be ruled out as contributors.

2. Lines 403–407. The suggestion that reduced perceived financial hardship during P1 reflects households “accumulating savings” during lockdown rests on a national average (consumption fell 7.1%) and is questionable for a low-income, socially vulnerable population, for whom the benefit increases described at lines 401–402 are a more plausible explanation. Please reconsider or temper this mechanism.

3. Lines 455–460. “this investment appears cost-effective” is not supported, as no cost or cost-effectiveness data were collected. Please soften to efficiency in terms of time and wasted trips, or remove the cost-effectiveness claim.

4. Throughout the Discussion and Conclusion. Provider engagement (supported by the qualitative data and the in-person delivery indicator) and participant engagement/reach (mixed: stable participation, declining in P3) are at times conflated under “engagement.” Please distinguish these clearly, since the evidence differs in strength.

5. Lines 376–378 and 481–482. Claims of “robust implementation” should be reconciled with the P3 decline in participation and in-person delivery. The data arguably show stability with weakening in later periods, which is a more nuanced picture than uniform robustness.

6. Lines 381–423. This section relies heavily on external Insee/Ipsos statistics to build a mechanistic narrative. While useful for context, the study cannot test these mechanisms; consider trimming and signposting more clearly which statements are study findings versus external context.

7. Lines 413–418. The food-insecurity figure (fresh fruit/vegetables, 27% in 2018 to 43% in 2023) lacks a citation; please add the source.

Limitations and strengths

1. Lines 475–492. The limitations are currently confined mainly to recall bias. Please expand to address: (a) the before/after design without a control group and the resulting inability to attribute changes to the crises rather than to secular or implementation-related trends; (b) the single-site focus (Lille only; Valenciennes excluded), limiting generalizability; (c) the dependence of the eligibility rate on prescreening/distribution practices; (d) multiple testing without adjustment; (e) qualitative analysis by a single coder and the secondhand nature of participants' reported views; and (f) unreported missing data and the denominator inconsistencies noted above.

2. Lines 478–481. The absence of Effectiveness and Maintenance outcomes is framed as consistent with RE-AIM's staged logic. This is reasonable, but it remains a limitation for conclusions about whether reach/engagement translated into benefit; please present it as such.

Conclusions

1. Lines 494–508. The conclusion is somewhat more optimistic than the data support and largely restates the Discussion. Please temper the claims of resilience to reflect the temporal heterogeneity (improvement in P1, decline by P3), and ensure conclusions about “engagement” specify provider versus participant engagement.

**********

what does this mean?). If published, this will include your full peer review and any attached files.

If you choose “no”, your identity will remain anonymous but your review may still be made public.

**Do you want your identity to be public for this peer review?** For information about this choice, including consent withdrawal, please see our Privacy Policy

Reviewer #1: **Yes:**  G.J. Hodson

Reviewer #2: **Yes:**  Dra. Araceli Guerra Martínez

Reviewer #3: **Yes:**  Renee Bargman

Reviewer #4: No

**********

[NOTE: If reviewer comments were submitted as an attachment file, they will be attached to this email and accessible via the submission site. Please log into your account, locate the manuscript record, and check for the action link "View Attachments". If this link does not appear, there are no attachment files.]

To ensure your figures meet our technical requirements, please review our figure guidelines: https://journals.plos.org/plosone/s/figures

You may also use PLOS’s free figure tool, NAAS, to help you prepare publication quality figures: https://journals.plos.org/plosone/s/figures#loc-tools-for-figure-preparation.

NAAS will assess whether your figures meet our technical requirements by comparing each figure against our figure specifications.

# Author comment: Author response to Decision Letter 1

**DOI:** 10.1371/journal.pone.0350372.r002

14 Aug 2026

Our point-by-point responses to the reviewers have been uploaded as a separate file.

Attachment Submitted filename: Reponses to Reviewers.pdf

# Peer review: Decision Letter 1

**DOI:** 10.1371/journal.pone.0350372.r003

**Authors:** Javier Fagundo-Rivera

21 Sep 2026

Reach and engagement in the ECAIL trial targeting socially disadvantaged families across the COVID-19 pandemic and cost-of-living crisis: a mixed-methods implementation study

PONE-D-26-23207R1

Dear Dr. Lioret,

We’re pleased to inform you that your manuscript has been judged scientifically suitable for publication and will be formally accepted for publication once it meets all outstanding technical requirements.

Within one week, you’ll receive an e-mail detailing the required amendments. When these have been addressed, you’ll receive a formal acceptance letter and your manuscript will be scheduled for publication.

An invoice will be generated when your article is formally accepted. Please note, if your institution has a publishing partnership with PLOS and your article meets the relevant criteria, all or part of your publication costs will be covered. Please make sure your user information is up-to-date by logging into Editorial Manager at Editorial Manager® and clicking the ‘Update My Information' link at the top of the page. For questions related to billing, please contact billing support.

If your institution or institutions have a press office, please notify them about your upcoming paper to help maximize its impact. If they’ll be preparing press materials, please inform our press team as soon as possible -- no later than 48 hours after receiving the formal acceptance. Your manuscript will remain under strict press embargo until 2 pm Eastern Time on the date of publication. For more information, please contact onepress@plos.org.

Kind regards,

Javier Fagundo-Rivera, PhD

Academic Editor

PLOS One

**Additional Editor Comments:**

Manuscript ID: PONE-D-26-23207R1

Title: "Reach and engagement in the ECAIL trial targeting socially disadvantaged families across the COVID-19 pandemic and cost-of-living crisis: a mixed-methods implementation study"

Dear Dr. Lioret,

I am pleased to inform you that your manuscript has been judged scientifically suitable for publication and will be formally accepted for publication in PLOS ONE once you have provided the remaining formal requirements listed below.

Before proceeding, I would like to explain the basis for this decision, since it differs from the recommendation of one of the reviewers involved in this revision round.

This revised manuscript was evaluated by one of the four reviewers who assessed the original submission; the other three reviewers were not available to provide a further assessment within a reasonable timeframe. The reviewer who did respond changed their recommendation from major revision to reject, on the basis that the aim and contribution of the study were not clear to them.

Having carefully weighed this recommendation against the manuscript itself, the authors' detailed response to the first round of reviews, and the assessments provided by all four reviewers in that first round, I concluded that rejection was not warranted. The reviewer's comments centered on the perceived purpose and length of the paper rather than on specific methodological or analytical shortcomings, and did not identify substantive flaws that would compromise the validity or contribution of the work. In my assessment, the manuscript makes a clear and meaningful contribution: it addresses an important implementation question regarding the reach and engagement of the intervention among socially disadvantaged families, with explicit attention to the structural and contextual barriers these families faced across the COVID-19 pandemic and the cost-of-living crisis.

I was also satisfied that the authors had responded comprehensively and appropriately to the major revisions requested by all four reviewers in the first round, and no unresolved substantive issues remained in the current version.

For these reasons, my decision reflects an overall editorial assessment of the revised manuscript and its review history, rather than the recommendation of a single reviewer considered in isolation.

Once we have confirmed that technical requirements have been met, your manuscript will be passed to our production department, and you will be notified when it is scheduled for publication. You will receive a proof of your article before final publication.

If you have any questions, please do not hesitate to contact us.

We look forward to publishing your manuscript in PLOS ONE.

Kind regards,

Javier Fagundo-Rivera, PhD

Academic Editor

PLOS ONE

Reviewers' comments:

Reviewer's Responses to Questions

**Comments to the Author**

Reviewer #3: All comments have been addressed

**********

2. Is the manuscript technically sound, and do the data support the conclusions?

Reviewer #3: No

**********

3. Has the statistical analysis been performed appropriately and rigorously?

Reviewer #3: I Don't Know

**********

4. Have the authors made all data underlying the findings in their manuscript fully available?

The PLOS Data policy

Reviewer #3: Yes

**********

5. Is the manuscript presented in an intelligible fashion and written in standard English?

Reviewer #3: Yes

**********

Reviewer #3: I continue to not understand the question that this study is answering. Why are they writing this paper?

**********

what does this mean?). If published, this will include your full peer review and any attached files.

If you choose “no”, your identity will remain anonymous but your review may still be made public.

**Do you want your identity to be public for this peer review?** For information about this choice, including consent withdrawal, please see our Privacy Policy

Reviewer #3: No

**********

# Editor report: Acceptance letter

**DOI:** 10.1371/journal.pone.0350372.r004

**Authors:** Javier Fagundo-Rivera

PONE-D-26-23207R1

PLOS One

Dear Dr. Lioret,

I'm pleased to inform you that your manuscript has been deemed suitable for publication in PLOS One. Congratulations! Your manuscript is now being handed over to our production team.

At this stage, our production department will prepare your paper for publication. This includes ensuring the following:

* All references, tables, and figures are properly cited

* All relevant supporting information is included in the manuscript submission,

* There are no issues that prevent the paper from being properly typeset

You will receive further instructions from the production team, including instructions on how to review your proof when it is ready. Please keep in mind that we are working through a large volume of accepted articles, so please give us a few days to review your paper and let you know the next and final steps.

Lastly, if your institution or institutions have a press office, please let them know about your upcoming paper now to help maximize its impact. If they'll be preparing press materials, please inform our press team within the next 48 hours. Your manuscript will remain under strict press embargo until 2 pm Eastern Time on the date of publication. For more information, please contact onepress@plos.org.

You will receive an invoice from PLOS for your publication fee after your manuscript has reached the completed accept phase. If you receive an email requesting payment before acceptance or for any other service, this may be a phishing scheme. Learn how to identify phishing emails and protect your accounts at https://explore.plos.org/phishing.

If we can help with anything else, please email us at customercare@plos.org.

Thank you for submitting your work to PLOS One and supporting open access.

Kind regards,

PLOS One Editorial Office Staff

on behalf of

Dr. Javier Fagundo-Rivera

Academic Editor

PLOS One
